# Transcriptional landscape of *Burkholderia pseudomallei* cultured under environmental and clinical conditions

**DOI:** 10.1099/mgen.0.000982

**Published:** 2023-04-05

**Authors:** Cin Kong, Rui-Rui Wong, Ahmad-Kamal Ghazali, Yuka Hara, Tengku Nurfarhana Tengku Aziz, Sheila Nathan

**Affiliations:** ^1^​ Department of Biological Sciences and Biotechnology, Faculty of Science and Technology, Universiti Kebangsaan Malaysia, Bangi, Selangor, Malaysia; ^2^​ Faculty of Health and Life Sciences, Inti International University, Nilai, Negeri Sembilan, Malaysia; ^‡^​ Present address: Division of Biomedical Sciences, University of Nottingham Malaysia, Selangor, Semenyih, Malaysia; ^§^​ Present address: Faculty of Biology, Medicine and Health, University of Manchester, Manchester, UK

**Keywords:** *Burkholderia pseudomallei*, comparative transcriptomics, biofilm, environmental niche, human plasma

## Abstract

*
Burkholderia pseudomallei *, a Gram-negative pathogen, is the causative agent of melioidosis in humans. This bacterium can be isolated from the soil, stagnant and salt-water bodies, and human and animal clinical specimens. While extensive studies have contributed to our understanding of *B. pseudomallei* pathogenesis, little is known about how a harmless soil bacterium adapts when it shifts to a human host and exhibits its virulence. The bacterium’s large genome encodes an array of factors that support the pathogen’s ability to survive under stressful conditions, including the host’s internal milieu. In this study, we performed comparative transcriptome analysis of *B. pseudomallei* cultured in human plasma versus soil extract media to provide insights into *B. pseudomallei* gene expression that governs bacterial adaptation and infectivity in the host. A total of 455 genes were differentially regulated; genes upregulated in *B. pseudomallei* grown in human plasma are involved in energy metabolism and cellular processes, whilst the downregulated genes mostly include fatty acid and phospholipid metabolism, amino acid biosynthesis and regulatory function proteins. Further analysis identified a significant upregulation of biofilm-related genes in plasma, which was validated using the biofilm-forming assay and scanning electron microscopy. In addition, genes encoding known virulence factors such as capsular polysaccharide and flagella were also overexpressed, suggesting an overall enhancement of *B. pseudomallei* virulence potential when present in human plasma. This *ex vivo* gene expression profile provides comprehensive information on *B. pseudomallei *’s adaptation when shifted from the environment to the host. The induction of biofilm formation under host conditions may explain the difficulty in treating septic melioidosis.

## Data Summary

The transcript reads of the *Burkholderia pseudomallei* UKMH10 cultured in different media have been deposited in the European Nucleotide Archive, project number PRJEB53338.

1. Transcript reads of *B. pseudomallei* UKMH10 cultured in human plasma have been deposited in the European Nucleotide Archive; accessions ERS12148041 and ERS12148042.
2. Transcript reads of *B. pseudomallei* UKMH10 cultured in soil extract medium have been deposited in the European Nucleotide Archive; accessions ERS12148045 and ERS12148046.

**Impact Statement**

*
Burkholderia pseudomallei *, a soil-dwelling bacterium, is the causative agent of melioidosis, a fatal infectious disease of humans and animals. The bacterium has a large genome spread over two chromosomes containing genes that encode for proteins with important roles for survival in diverse environments as well as in the infected host. *B. pseudomallei *’s ability to survive in a harsh, low-nutrient soil or water environment and subsequently transition and adapt in the host environment is a key question that should be resolved to identify potential drug or inhibitor targets. In this study, we explored the gene expression profiles of *B. pseudomallei* grown in media mimicking the soil environment and bacteria cultured in blood plasma to mimic the human milieu. A significant number of genes encoding for proteins implicated in biofilm development were modulated in *B. pseudomallei* grown in human plasma. This suggests that the bacteria produce biofilm in an infected host to defend themselves against the immune response and antimicrobial treatment. Also of interest was the observation that the expression of known virulence factors was positively modulated under host-mimic conditions. Nonetheless, *B. pseudomallei* grown in both soil medium and plasma were almost equally virulent in infected mice. The transcriptome profile and the virulence assessment propose that *B. pseudomallei* activates a different set of genes once it transitions from soil into the host that explains its ability to survive and demonstrate pathogenicity.

## Introduction

*Burkholderia pseudomallei *, the causative agent of melioidosis, is a Gram-negative soil saprophyte that infects humans and animals. Melioidosis is prevalent globally, but predominantly in Southeast Asia and northern Australia, where high mortality rates are noted [1, 2]. As the majority of infection is thought to occur in rural tropical locations where a lack of suitable resources hampers accurate diagnosis, a modelling study has proposed that the reported rates of melioidosis could be up to 165 000 cases of melioidosis and 89 000 deaths worldwide per year [1]. *B. pseudomallei* occupies various natural ecological niches, particularly within moist tropical soils, rice paddy fields and stagnant water in endemic areas [3, 4]. Humans are infected by *B. pseudomallei* present in contaminated soil or water bodies via direct contact through percutaneous inoculation, ingestion or inhalation, which is exacerbated after the occurrence of heavy monsoonal rains, cyclones or typhoons [5]. The clinical manifestations of melioidosis vary from acute to chronic infections, including pneumonia, septicaemia, skin ulcers and disseminated abscesses in organs [6, 7].

Knowledge of how a pathogen adapts to different environments by modulating its gene expression is key to understanding the molecular mechanisms underlying infection and pathogenesis. Genome-scale platforms have been used to investigate global gene expression changes in bacteria exposed to different conditions. Previous microarray and comparative transcriptomic studies of *Burkholderia cenocepacia* grown under conditions that mimic a natural pathogenic niche (human sputum) versus the soil environment revealed numerous putative virulence genes involved in chronic respiratory infection that can serve as potential therapeutic targets [8–10]. Gene expression studies to compare the transcriptome profile of environmental and clinical isolates of *Vibrio* spp. exposed to human serum relative to its natural habitat provided new knowledge on genes required for bacterial fitness and genes that allow a free-living environmental bacteria to emerge as a human pathogen [11, 12]. The availability of *B. pseudomallei* microarrays has enabled *in vitro* gene expression studies to be performed at the genome level. Using whole-genome tiling microarrays, Ooi *et al*. reported the transcriptional landscape of *B. pseudomallei* under 80 different environmental and genetic conditions and identified several key physiological processes, as well as genes involved in quorum sensing and *in vivo* infections [13]. The microarray-derived transcriptome profile of *B. pseudomallei* infecting human macrophage cells also revealed the modulation of genes important in intracellular survival and adaptation during host cell infection [14]. RNA sequencing was first used to compare the expression of *B. pseudomallei* isolated from five cystic fibrosis patients and identified differential expression patterns of loci for antibiotic resistance and virulence factors [15].

Despite the availability of epidemiological reports on the geographical distribution of melioidosis, not much is known about the underlying mechanisms that contribute to the emergence of *B. pseudomallei* from harmless soil bacteria to highly virulent bacteria that cause fatal infections in humans. Additionally, current knowledge on the role of pathogenic factors and specific mechanisms of survival and virulence in the soil-dwelling *B. pseudomallei* is still a limiting factor in the design of new therapeutics for melioidosis.

In the present study, we employed RNA sequencing technology to assess the differential gene expression of *B. pseudomallei* cultured in soil (native) and human plasma (clinical) environments to understand why a soil-dwelling bacterium turns pathogenic. We compared the transcriptional response in these two distinct conditions mimicking the natural habitat and clinical environment at the genome level to identify putative factors involved in virulence, survival and adaptation to the host. We also determined the virulence of bacteria grown in soil medium and plasma using a mouse infection model and compared the expression profile of genes associated with known virulence factors under both conditions. Overall, this study provides insights into the transcriptional response of *B. pseudomallei* grown under different niches and has advanced our understanding of differential expression of virulence factors that turn *B. pseudomallei* into a deadly pathogen when it shifts from its natural reservoir to a human host.

## Methods

### Bacterial strain and culture media

*B. pseudomallei* strain UKMH10, a clinical isolate from a melioidosis patient, was used in this study. The phenotypic characterization of UKMH10 was described in a previous study [16]. A stock culture was obtained from the Pathogen Laboratory, Faculty of Science and Technology, Universiti Kebangsaan Malaysia and stored at −80 °C. The bacteria were routinely cultured on Ashdown’s agar [17] at 37 °C. Soil extract medium (SEM) was prepared as previously described [8]. Briefly, 400 g of soil taken from a depth of 0–20 cm was sieved and mixed in 1 l of distilled water, followed by sterilization at 121 °C for 15 min. The soil–water mixture was centrifuged (5 min at 2000 ***g***) and the supernatant filtered with a 0.45 µm syringe filter to remove any particulates. Soil medium (10 % soil extract) was prepared by diluting the soil extract in sterilized distilled water to a 1 : 10 dilution and glucose was supplemented to a final concentration of 3 mM as the carbon source for the bacteria. Fresh whole blood was obtained from National Blood Bank Malaysia in EDTA-treated tubes. Cells were removed from human plasma by centrifugation for 10 min at 2000 ***g*** at 4 °C. The plasma was stored at −80 °C until required.

### Bacterial cultivation and total RNA preparation

A single colony of *B. pseudomallei* UKMH10 was inoculated into fresh Luria–Bertani (LB) broth (Pronadisa, Spain) and grown overnight at 37 °C, with shaking at 250 r.p.m. The overnight bacterial culture was further sub-cultured into human plasma and soil extract medium at a 1 : 50 dilution. To mimic the soil environment, SEM inoculated with *B. pseudomallei* was incubated at 30 °C, whilst the human plasma inoculum was incubated at 37 °C to mimic the human body temperature. When the absorbance reading of the bacterial cultures at 600 nm (OD_600_) reached 0.5, cells were harvested by centrifugation at 4000 ***g*** at 4 °C. Total RNA was extracted using TRIzol Reagent (Invitrogen Life Technologies, USA), purified with Qiagen’s RNeasy Mini kit and on-column *DNase*I digestion was performed to remove any residual DNA. The concentration and quality of all DNA-free RNA samples were analysed using a NanoDrop 1000 Spectrophotometer (Thermo Scientific, USA) and the RNA integrity was assessed using an Agilent 2100 Bioanalyzer.

### RNA sequencing and mapping of Illumina reads

RNA sequencing was conducted on the Illumina HiSeq 4000 sequencing platform (Illumina, USA) using a sequencing protocol based on the manufacturer’s instructions. The Illumina TruSeq kit (Illumina, USA) was used to prepare two biological replicate RNA-seq libraries. The libraries were sequenced using a 150 bp paired-end protocol to a sequencing depth of 90 million reads. The quality of the sequence generated was assessed using FastQC v0.10.1 (http://www.bioinformatics.bbsrc.ac.uk/projects/fastqc) and preprocessed to remove low-quality reads using the FASTX-Toolkit (http://hannonlab.cshl.edu/fastx_toolkit/) with parameters set to remove standard Illumina sequencing adapters. The preprocessing parameters were set based on a *Q* value >20 and a minimum read length of 30 bp. Orphan reads within the preprocessed reads were removed using the Python script and only paired reads were used in the analysis. All sequences generated have been deposited at The European Nucleotide Archive (ENA) with the project number PRJEB53338.

After the preprocessing was completed, the reads were mapped to the *B. pseudomallei* strain K96243 genome sequence (NCBI RefSeq NC_006350 and NC_006351) using the alignment tool TopHat v2.02 and its default parameters [18]. Samtools was used to convert the resulting SAM files to BAM files and to sort the BAM files [19]. Overall over 88 % of preprocessed reads were successfully mapped to the *B. pseudomallei* K96243 reference genome. Differential expression between transcripts of *B. pseudomallei* cultured in blood plasma versus soil extract media was performed using the Cuffdiff tool, a part of the Cufflink package version 2.02 [20] Transcripts with log_2_ fold change >2 or <−2 and a q-value of <0.05 were considered to be differentially expressed transcripts.

### Hierarchical clustering

Selected data were organized by hierarchical clustering with the web-based software Cluster 3.0 [21]. The clustering algorithm is based on an uncentred correlation metric with average linkage clustering and visualized using Java Treeview V1.1.3.

### Gene ontology and gene functional enrichment

Functional classifications were performed based on Comprehensive Microbial Resources (CMR) annotations (www.cmr.jcvi.org). Gene functional enrichment analysis was performed against the DAVID Bioinformatics Resources 6.7 database (https://david.ncifcrf.gov/) using Fisher’s exact test with Benjamini–Hochberg multiple testing correction (*P*<0.05).

### Quantitative RT-PCR (qRT-PCR)

qRT-PCR was performed with *DNase*I-treated total RNA using the iScript One-Step qRT-PCR kit with SYBR Green according to the manufacturer’s instructions (Bio-Rad Laboratories, USA). A panel of six genes upregulated and six genes downregulated in human plasma as compared to soil medium was selected for validation of the RNA sequencing data. The forward and reverse primers used were designed using the Realtime PCR Tool (Integrated DNA Technologies; https://www.idtdna.com/scitools/Applications/RealTimePCR/) and are listed in Table S1 (available in the online version of this article).

Specificity of amplification was confirmed by melt curve analysis after amplification of the products. Briefly, 20 µl reactions of 20 ng cDNA and 10 pmol of each primer were assembled according to the manufacturer’s instructions using the QuantiNova kit (Qiagen, Germany). Reactions were performed on the Bio-Rad CFX96 Real-Time PCR Detection System (Bio-Rad Laboratories, USA) using a programme of 95 °C for 2 min followed by 39 cycles of 95 °C for 5 s and 60 °C for 30 s. Two biological samples were run with three technical replicates, no template controls and no reverse transcriptase controls. The average cycle threshold (ΔΔ*C*_T_) values were normalized to a housekeeping gene (*B. pseudomallei recA* gene) where ΔΔ*C*_T_=*C*_T_ target gene – *C*_T_ *recA* gene. The mRNA expression level of genes of interest was computed using the CFX Manager software (Bio-Rad Laboratories, USA) on the average normalized *C*_T_ value.

### Crystal violet biofilm assay

An overnight culture of *B. pseudomallei* UKMH10 was diluted 1 : 50 in human plasma and soil medium and incubated at 37 and 30 °C, respectively. At the end of the incubation, the bacterial density was adjusted to OD_600_=1.0 using a spectrophotometer to obtain a standardized inoculum. For the biofilm quantification assay, 200 µl of bacterial suspension grown in each medium was added into 18 wells of a sterile 96-well flat bottom cell culture plate (Greiner Bio-One, Germany) and the plate was incubated at 37 °C for 48 h under static growth to allow the formation of biofilm. Uninoculated plasma and soil medium were included as the blank controls. After 48 h incubation, the wells were washed three times with 1× phosphate buffer to remove non-adherent bacterial cells, fixed with 99 % (v/v) methanol for 15 min and left to air-dry at room temperature. The wells were then stained with 200 µl of filtered crystal violet for 5 min. The excess crystal violet stain was washed with sterile distilled water and the wells were air-dried in a biosafety cabinet. The crystal violet bound to the bacterial cells was solubilized with 200 µl of 95 % (v/v) ethanol and the released stain was measured using a microplate reader (Tecan, Switzerland) at 630 nm. For a qualitative assay, 2 ml of bacterial suspension cultured in human plasma and soil medium was dispensed into test tubes and the cells were stained with 2 ml of crystal violet. Without solubilizing the crystal violet, a photograph of the stained biofilm formed on the wall of the test tube was taken using a digital camera. Three independent assays were carried out.

### Scanning electron microscopy (SEM) analysis of biofilm formation

An overnight culture of *B. pseudomallei* UKMH10 was diluted 1 : 50 in human plasma and soil medium and incubated as described above. Bacterial density was adjusted to OD_600_=1.0. For each culture, 4 ml of bacterial suspension was added to a six-well plate with a 10×10 mm glass cover slip placed in the middle of each well. The plate was incubated at 37 °C for 48 h under static conditions to allow the formation of biofilm on the surface of the glass cover slips. Subsequently, the cover slips with bacterial biofilms were fixed with 4 % (v/v) glutaraldehyde for 12–24 h at 4 °C. The samples were washed three times in phosphate buffer, dehydrated through a graded ethanol series, dried in a critical-point drying apparatus with liquid carbon dioxide, sputter-coated with gold and viewed under a LEO 1450VP scanning electron microscope (Zeiss, Germany) at the Electron Microscopy Unit, Universiti Kebangsaan Malaysia.

### Mice survival assay

Female BALB/c mice, aged 6–8 weeks old, were obtained from the Animal House Facility, Universiti Kebangsaan Malaysia. Animals were maintained under specific-pathogen-free conditions in a positive pressure environment at 20–25 °C, subjected to a 12 h light/dark cycle and provided with pelleted protein-enriched diet and water *ad libitum. B. pseudomallei* UKMH10 was cultured in human plasma or soil medium as described above. Mice were challenged intraperitoneally with ~1×10^6^ c.f.u. of *B. pseudomallei* in 200 µl of phosphate-buffered saline (PBS) and mice survival was monitored. The uninfected control group was injected with 200 µl of PBS.

### Virulence factor analysis

The set of differentially expressed genes was submitted to the Virulence Factor Database (VFDB) (http://www.mgc.ac.cn/VFs/) [22] to search for the presence of known *B. pseudomallei* K96243 virulence factors expressed under both culture conditions.

### Statistical analysis

Statistical analyses were performed using the Stata v12.0 statistical software package (StataCorp LP, USA). Student’s *t*-test was used to assess the significance in difference between biofilm production. All data in this study are presented as mean±standard deviation. Differences were considered statistically significant at a *P* value <0.05.

## Results

### Global transcriptional profiles of *B. pseudomallei* cultured in plasma and soil

**Fig. 1.**
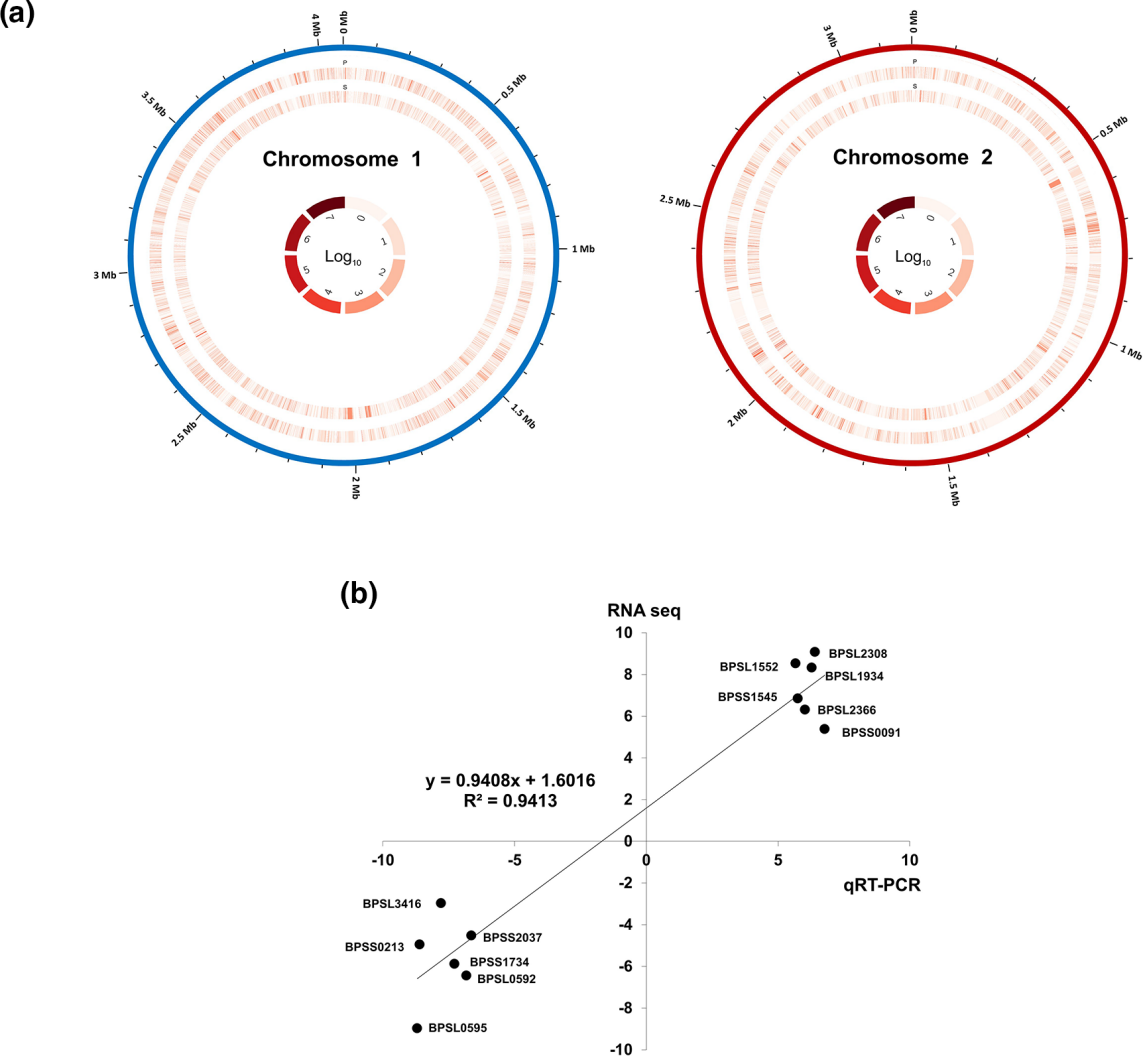
Circular map depicting expression of *B. pseudomallei* transcripts in f.p.k.m. mapped to the *B. pseudomallei* K96243 reference genome. The inner ring indicates the f.p.k.m. for soil sample whilst the outer ring is for plasma sample. (**b**) Plot showing positive correlation between the results of qRT-PCR and RNA-seq.

To delineate changes in the transcriptional landscape of *B. pseudomallei* when it shifts from its native (soil) to a clinical (infection) environment, the bacteria were cultured in soil extract medium [8] and human plasma to mimic the natural soil niche of the bacteria and clinical niche in infected humans, respectively. The *B. pseudomallei* strain used in this study is the human isolate *B. pseudomallei* UKMH10 [16]. *B. pseudomallei* was grown in soil extract medium at 30 °C to represent the typical tropical rainforest soil temperature or in human plasma at 37 °C prior to total RNA isolation and purification. Two independent RNA samples for each condition were subjected to sequencing on the Illumina HiSeq platform and the clean sequencing reads were mapped to the *B. pseudomallei* K96234 reference genome. Over 90 % of the total reads for all replicate samples were successfully mapped to the reference genome (Table 1). The mapped clean reads were then converted to gene expression units, Fragments per kilobase of exon per million fragments mapped (f.p.k.m.). The expression cut-off was set at f.p.k.m. >0.1 and differential expression profiles of *B. pseudomallei* UKMH10 cultured under both plasma and soil conditions were plotted on a circular map according to the gene location on the *B. pseudomallei* K96243 chromosomes 1 and 2 (Fig. 1a).

**Table 1.** Analysis of *B. pseudomallei* UKMH10 transcriptome sequencing reads mapped to the reference genome of *B. pseudomallei* K96243 genome

Samples	Plasma 1	Plasma 2	Soil 1	Soil 2
Total no. of clean reads	11 957 128	13 508 888	10 138 252	10 502 632
Total no. of reads mapped	11 195 531	12 620 137	9 394 392	9 670 540
Percentage of total reads mapped to K96243 (%)	93.63	93.42	92.66	92.07
Total no. of mapped paired reads	10 825 745	12 191 988	9 053 905	9 302 681
Total no. of mapped singletons	369 786	428 149	340 487	367 859
Total no. of unmapped reads	761 597	888 751	743 860	832 092
Number of uniquely mapped reads	11 019 093	12 508 931	9 310 512	9 526 604
Number of multiple mapped reads	938 035	999 957	827 740	976 028
Reads mapped to CDSs	5399	5447	5391	5439
Percentage of CDSs mapped (%)	93.07	93.90	92.93	93.76

We then performed independent quantitative real-time PCR (qRT-PCR) analysis to validate the RNA-seq-derived expression data. A subset of genes representing various functional categories were selected for qRT-PCR (Table S1). The relative expression levels of six upregulated genes and six downregulated genes were analysed and compared using RNA extracted from three different batches of *B. pseudomallei* culture grown in soil medium and plasma. A strong positive correlation was noted between the qRT-PCR and RNA-seq expression for all 12 genes tested with an *R* ^2^ value of 0.9413 (Figs 1b and S1).

**Fig. 2.**
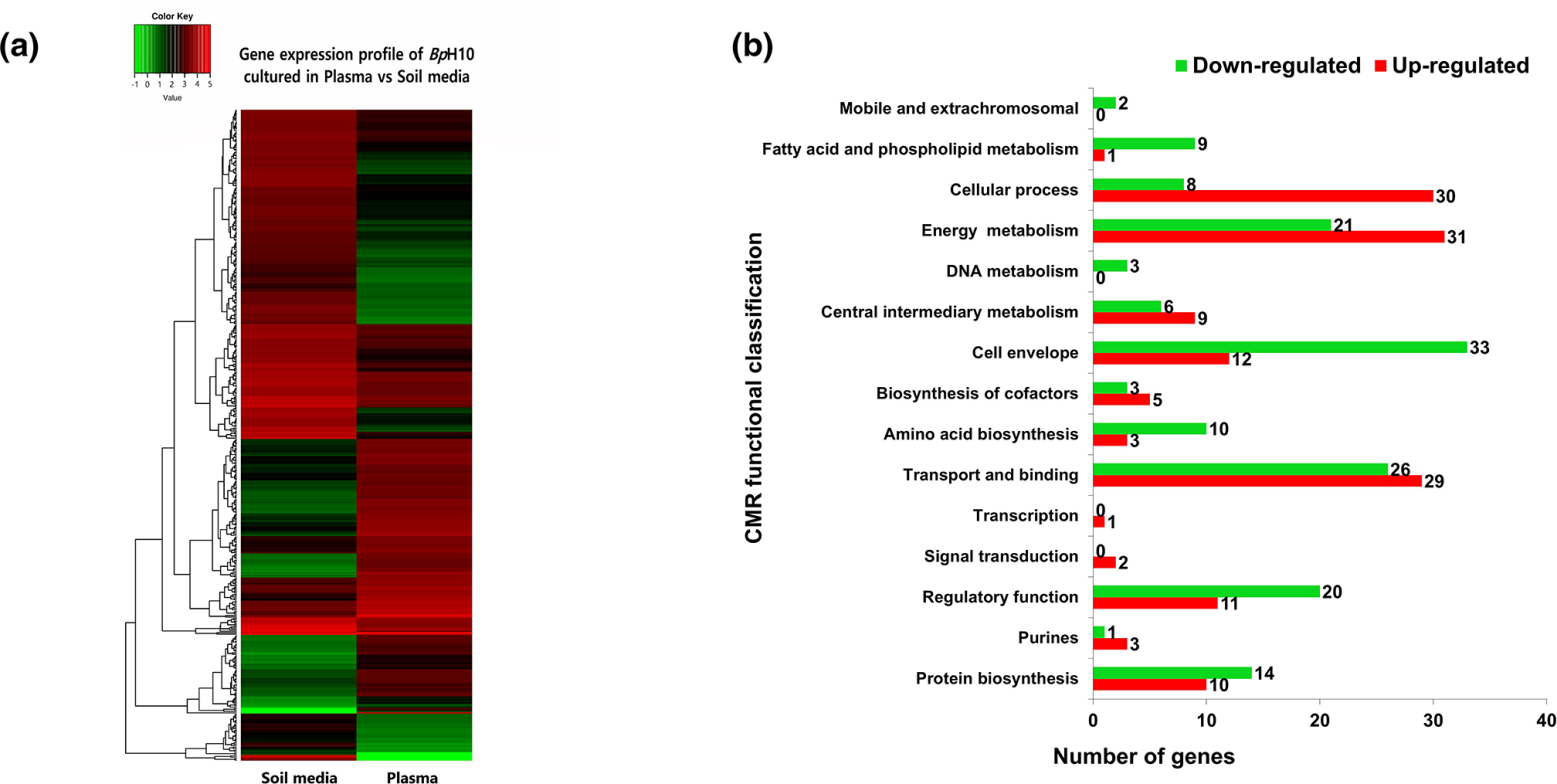
Hierarchically clustered expression profile (rows) for 455 *B. pseudomallei* UKMH10 genes differentially expressed when the bacteria were cultured in human plasma compared with soil medium. Data from two independent experiments (columns) are shown. (**b**) Functional classification of differentially regulated genes between plasma- and soil-grown *B. pseudomallei* using Comprehensive Microbial Resources (CMR).

A total of 455 *B. pseudomallei* genes were significantly expressed in human plasma as compared to soil medium (cut-off of log_2_ fold change >2 and <−2, *Q* value <0.05). Of these, 181 genes were upregulated and 274 genes were downregulated in bacteria cultured in human plasma relative to soil (Fig. 2a). A comprehensive list of genes differentially expressed under the plasma versus soil conditions is available in Table S2. The biological functions of significantly modulated genes were assigned based on the CMR annotation. Of note, more than a third of the modulated genes were grouped as genes with unknown function (6 %), hypothetical proteins (19.5 %) and unclassified genes (8 %). Functional classification of up- and downregulated genes showed that most of these genes encode core functions such as cellular processes, energy metabolism, cell envelope, transport and binding, regulatory functions and protein biosynthesis (Fig. 2b). The distinct functional profiles observed between *B. pseudomallei* grown in soil medium and plasma suggest that the gene expression patterns are unique to the specific environmental cues encountered by the bacteria.

From the differential expression analysis between the two conditions, genes that were induced in human plasma encoded for proteins that were mainly involved in cellular processes and energy metabolism, whilst most of the genes encoding cell envelope, fatty acid and phospholipid metabolism, amino acid biosynthesis and regulatory function proteins were downregulated in plasma as compared to soil medium (Fig. 2b). Interestingly, the repression of genes encoding proteins required for envelope biosynthesis, amino sugar and nucleotide sugar metabolism, and fatty acid biosynthesis were also observed in *B. pseudomallei-*infected macrophage cells [14]. *B. pseudomallei* modulates the bacterial surface structures such as envelope proteins to avoid host immune system recognition during survival inside host cells [23].

### Genes overexpressed in *B. pseudomallei* grown in plasma encode for proteins involved in various core functions

**Fig. 3.**
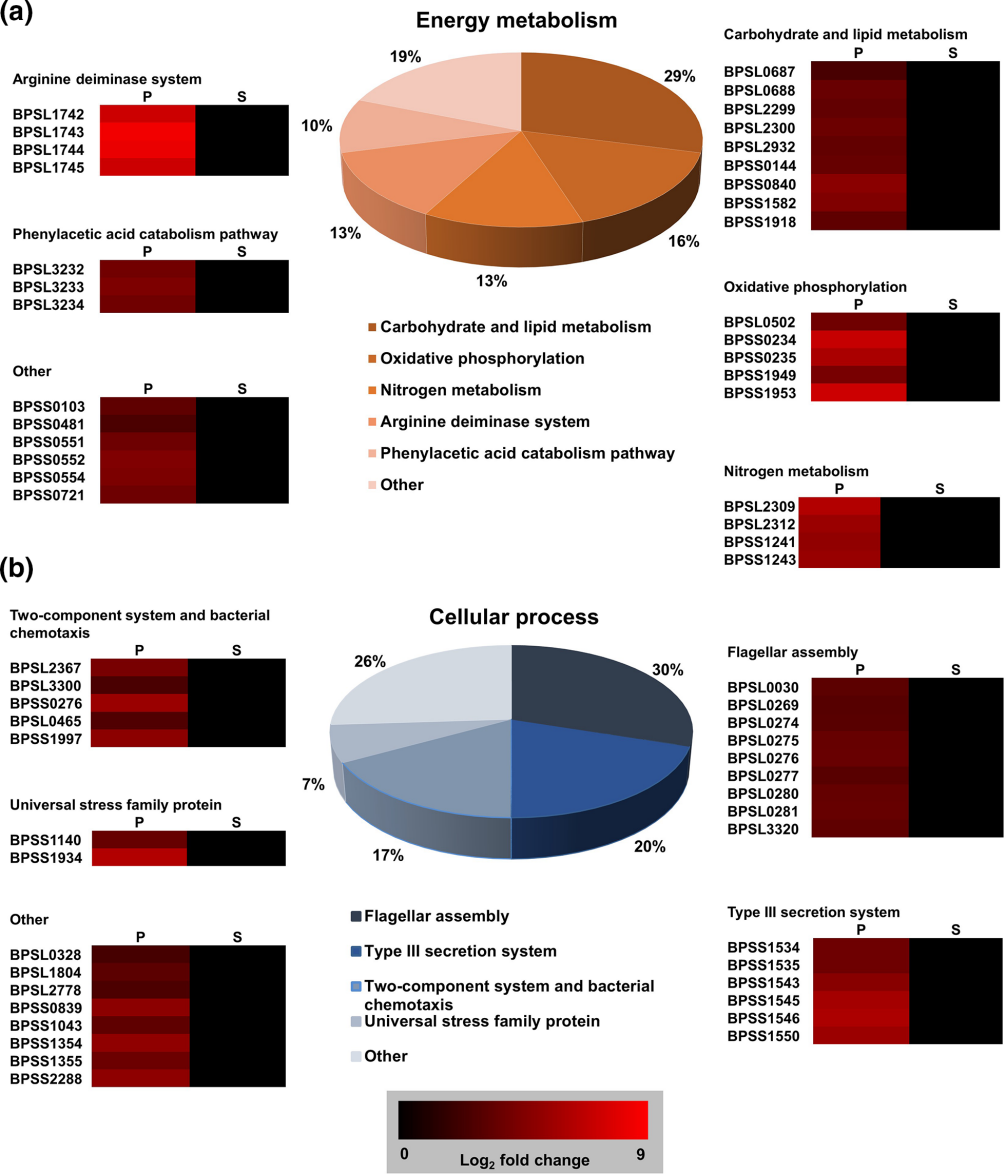
Functional enrichment based on DAVID for genes overexpressed in blood plasma (**p**) relative to soil medium (**s**). The pie charts represent genes involved in (**a**) energy metabolism and (**b**) cellular processes. The heat maps indicate gene expression in f.p.k.m.

The two main functional categories of genes overexpressed in bacteria grown in plasma were energy metabolism [31 genes (17 %)] and cellular processes [30 genes (16.6 %)]. For energy metabolism, 29 % of the genes fell into the category of carbohydrate and lipid metabolism and 16 % of the genes encoded proteins that play a role in oxidative phosphorylation (Fig. 3a). Among the nine carbohydrate and lipid metabolism genes, *bpsl2299*, *bpsl2300*, *bpss0804* and *bpss1918* encode for proteins involved in glycolysis or gluconeogenesis, implying that the bacteria undergo active cellular metabolic processes when present under infection conditions by oxidizing the nutrients available in plasma to produce energy (Table S3).

In addition, we also observed that the genes coding for arginine deiminase enzymes (*bpsl1742*, *bpsl1743*, *bpsl1744* and *bpsl1745*) were among the most highly induced genes in the energy metabolism category (log_2_ fold change of 7–8.5) (Fig. 3a). The four genes correspond to *arcD*, *arcA*, *arcB* and *arcC*, which are arranged in sequence and form an operon that makes up the arginine deiminase system in *B. pseudomallei* [24]. Generally, this system functions as a secondary metabolic pathway to metabolize arginine to produce ATP, carbon dioxide and ammonia. In *B. pseudomallei *, the arginine deiminase system is induced in response to the presence of arginine, acidic environments and low oxygen concentrations [24].

For the functional category of cellular process, of the 30 genes positively regulated in bacteria cultured in plasma, a third are involved in flagella assembly while 20 % are genes that make up the type III secretion system (T3SS) (Fig. 3b). The nine flagella assembly-associated genes induced in plasma are *bpsl0030*, *bpsl0269*, *bpsl0274*, *bpsl0275*, *bpsl0276*, *bpsl0277*, *bpsl0280*, *bpsl0281* and *bpsl3320*, with log_2_ fold changes ranging from 3 to 3.7 (Fig. 3b, Table S3). However, we noted that the *B. pseudomallei fliC* (encoding flagellin) gene was not significantly modulated in plasma or soil. Flagella are important for bacterial movement and adherence. *B. pseudomallei* flagella are a key virulence factor involved in macrophage invasion and aflagella mutant bacteria are avirulent in a mouse model of infection [25]. *B. pseudomallei* were previously observed to be highly motile at 37 °C and increasing the temperature to 40 or 42 °C renders them less motile. Reduced motility was also observed in bacteria cultured at 30 °C [26]. In the present study, the bacteria cultured in plasma was incubated at 37 °C, whilst the soil culture was kept at 30 °C before the extraction of RNA from bacterial cells. Thus, the induction of flagella-associated genes might be regulated by a change in environmental temperature.

A subset of genes, including *bpss1534* (BsaZ), *bpss1535* (BsaY), *bpss1543*, *bpss1545* (BsaQ), *bpss1546* (BsaN) and *bpss1550*, that play a role in the T3SS were also upregulated in bacteria cultured in plasma (Fig. 3b, Table S3). The human body is generally viewed as a harsh environment for pathogens and in order to adapt and survive in such an environment, Gram-negative bacteria, including *B. pseudomallei *, use secretion systems to transport proteins into the surrounding environment. Three T3SSs can be found in *B. pseudomallei *, namely T3SS-1, T3SS-2 and T3SS-3. T3SS-3 proteins are encoded by the cluster of genes corresponding to *bpss1516–bpss1552*, and all six T3SS genes upregulated in bacteria grown in plasma are located in the T3SS-3 cluster.

### Enhanced biofilm production by *B. pseudomallei* cultured in plasma

**Fig. 4.**
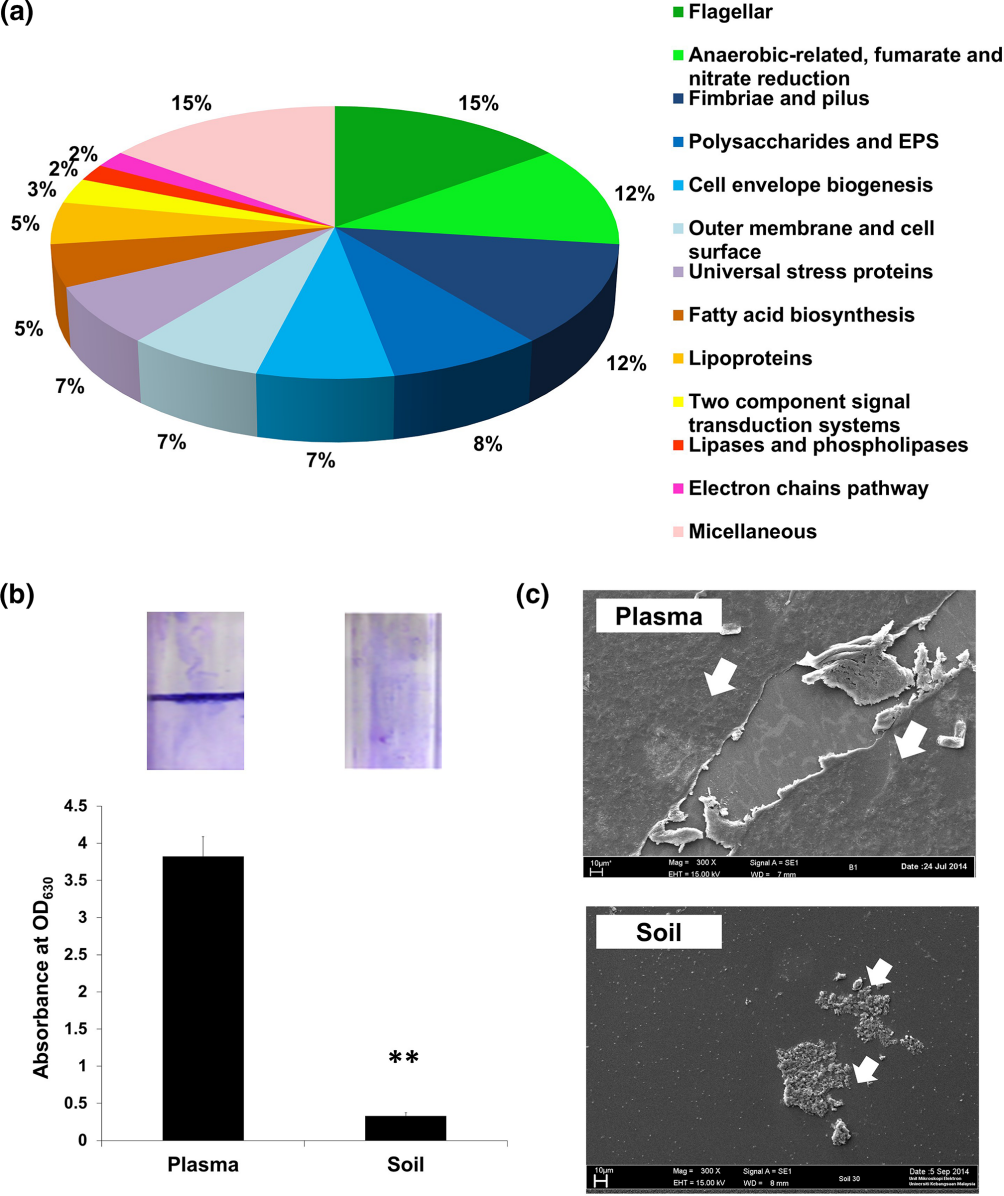
Distribution of biofilm-related genes based on functional categories. (**b**) Biofilm formation was detected using crystal violet staining in test tubes, followed by a quantitative measurement of biofilm at 630 nm. Biofilm formation was significantly lower (*P*<0.01) in *B. pseudomallei* grown in soil culture relative to human plasma. (**c**) SEM micrographs of *B. pseudomallei* grown in human plasma and soil.

Taweechaisupapong *et al*. reported that *B. pseudomallei* biofilms do not play a significant role in the disease process [27]. However, we subsequently showed that *B. pseudomallei* that produced a larger amount of biofilm were more virulent in both mouse and nematode models of infection [28]. When we compared the genes that were upregulated in plasma to our biofilm-associated gene list [28], almost a quarter (43 out of 181) of the genes overexpressed under plasma growth conditions were present in the list of genes proposed to have important roles in biofilm formation. These genes were categorized into the following major functional groups: flagella (15 %), anaerobic-related, fumarate and nitrate reduction (12 %), fimbriae and pilus (12 %), as well as polysaccharide and extrapolymeric substances (EPSs) (8 %) (Fig. 4a).The role of flagella in biofilm formation is to attach the free-living bacteria to the surface as the first step in biofilm production. A *B. pseudomallei fliC* (gene encoding flagellin) mutant produces less biofilm than the wild-type [29]. Similarly, fimbriae and pilus are also important in bacterial motility and *B. pseudomallei* biofilm attachment [28].

To confirm that *B. pseudomallei* grown in blood plasma do indeed produce more biofilm compared to the bacteria grown in a soil-mimic environment, we stained both groups of bacterial cells with crystal violet as well as observing the cells by scanning electron microscopy (SEM). Following 48 h growth under static conditions in the respective (plasma/soil) media, we noted that a significantly lower level of biofilm was formed in *B. pseudomallei* cultured in soil compared to the bacteria cultured in human plasma (*P*<0.01) (Fig. 4b). Furthermore, when the morphology of the biofilm architecture formed was visualized using SEM, the image showed a thick uniform layer of biofilm for bacteria grown in plasma (Fig. 4c). In contrast, a negligible amount of biofilm cells was observed for the soil culture (Fig. 4c). The observation of significant biofilm formation using both the crystal violet biofilm assay and SEM validated the upregulation of a significant number of biofilm-related genes seen for *B. pseudomallei* cultured in plasma.

### Growth in plasma induced the expression of *B. pseudomallei* virulence factor-encoding genes

**Fig. 5.**
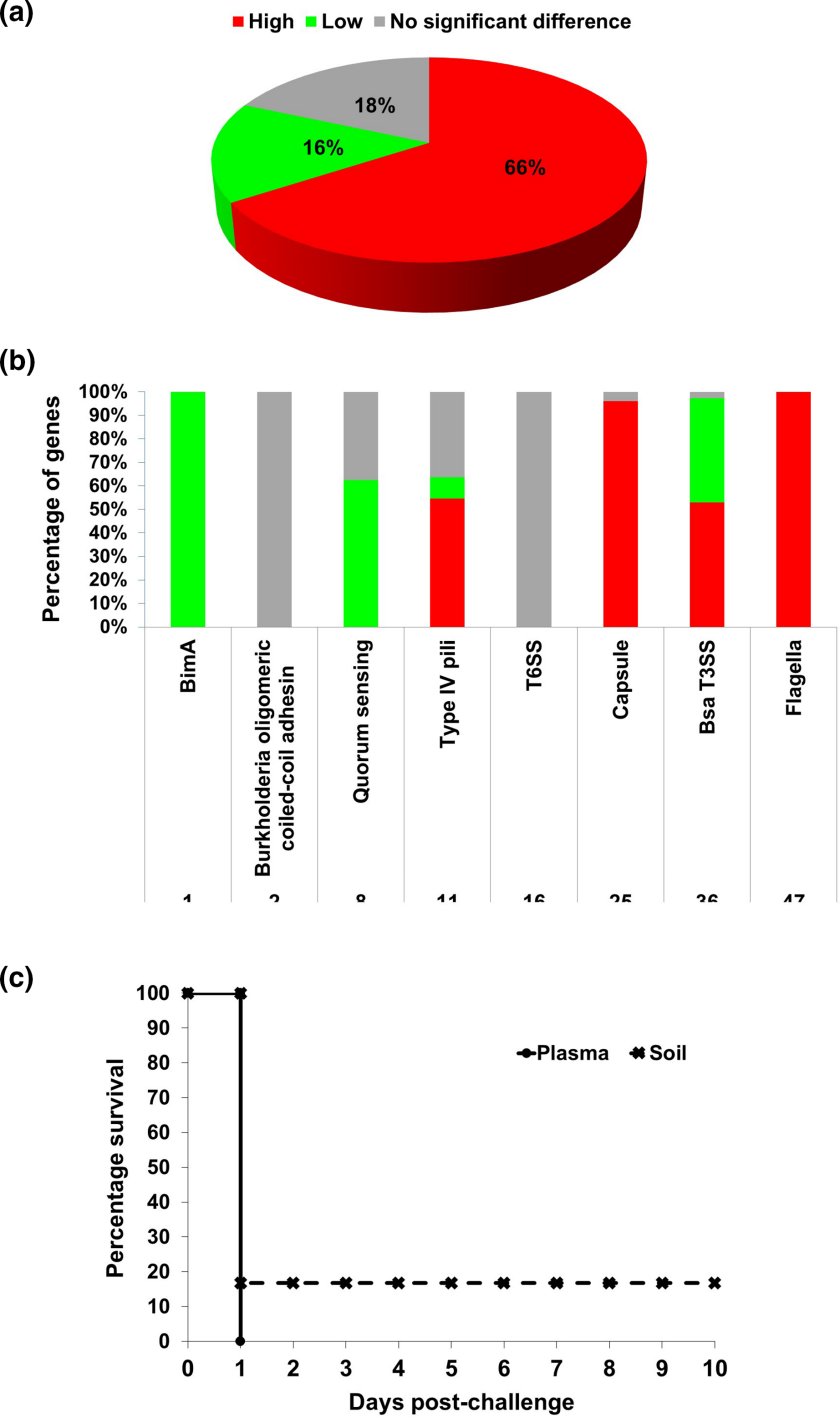
Overall expression of virulence-associated genes for *B. pseudomallei* grown in blood plasma and soil. The whole transcriptome profile was compared against the Virulence Factor Database (VFDB) and differentially regulated factors were identified. (**a**) Pie chart showing that 66 % of virulence genes were upregulated in blood plasma and 16 % in soil, whilst 18 % of them remained unchanged across the different growth media. (**b**) Bar chart showing the number and percentage of genes associated with specific *B. pseudomallei* virulence functions. (**c**) Kaplan–Meier survival analysis of BALB/c mice infected with *B. pseudomallei* grown in blood plasma and soil. Six mice from each group were challenged intraperitoneally with 1×10^6^ c.f.u. and their survival was monitored throughout a period of 10 days. Killing kinetics of both *B. pseudomallei* grown in soil sample and blood plasma was not significantly different (logrank test; *P*-value=0.317311).

A number of important *B. pseudomallei* virulence factors that contribute to the bacteria’s intracellular lifestyle and pathogenesis have been identified and characterized [30]. In this study, we were interested to establish if any of the previously described or possibly even novel *B. pseudomallei* virulence factor-encoding genes were differentially regulated when the bacteria are cultured in human plasma or soil media. We compared our transcriptome profiles against the Virulence Factors Database (VFDB) and 146 genes were identified. Among these 146 virulence genes, 96 genes (66 %) were upregulated when the bacteria were grown in plasma while 23 genes (16 %) were downregulated (Fig. 5a). We noted that among the genes positively regulated when *B. pseudomallei* were exposed to plasma were genes encoding flagella and capsular polysaccharide (CPS), while quorum sensing-associated genes were downregulated under the same condition (Fig. 5b). CPSs are cell surface exposed polysaccharides that have an essential role in *B. pseudomallei* virulence. CPSs block the deposition of complement factor C3b onto the bacteria, thus preventing phagocytosis by host immune cells [31, 32]. Consistent with the present study, expression of CPS I was previously reported to be triggered in response to human serum [32]. Hence, the shift from soil to host most likely triggers CPS synthesis whereby the presence of the CPS protects the bacteria from lysosomal defensins and cationic peptides and enables the pathogen to survive in the infected human.

Interestingly, all 47 known flagella genes were upregulated in *B. pseudomallei* grown in blood plasma (Fig. 5b). However, it is unclear why the bacteria would need to express all flagella-associated genes under host-mimic conditions. Flagella are required for bacterial attachment, a key step in the initial stage of biofilm formation [29, 33]. Likewise, type IV pili genes, which are similarly involved in bacterial attachment, were also among the genes overexpressed under plasma media growth conditions [33]. In contrast, none of the eight quorum sensing-related genes were upregulated in blood plasma. This is surprising, as quorum sensing is known to regulate the production of CPS, flagella and biofilm formation. For the T3SS-3-associated genes, we noted that all the components of the T3SS-3 delineated as virulence factors of *B. pseudomallei *, including the export apparatus (BsaZ), inner membrane ring (BsaM) and needle component (BsaL), were collectively upregulated. Unlike the structural genes, downregulated expression was noted in most of the regulator and effector genes, including *BopA* and *BopC*. Interestingly, expression of T6SS-5 (type VI secretion system 5) genes was unchanged between blood plasma and soil media. The T6SS-5 (also named cluster 1 T6SS) is required for the formation of multinucleate giant cells (MNGCs), although its mechanism is still unknown. Furthermore, T6SS-5 is crucial for intracellular survival and multiplication of *B. pseudomallei* upon host entry [34, 35]. However, blood plasma contains coagulants, albumin and globulin and is devoid of any cells. Under our experimental conditions, *B. pseudomallei* will not be involved in intracellular infection, hence there is limited requirement for the bacteria to overexpress T6SS-5.

To address the question of whether differential expression of these virulence genes affects *B. pseudomallei* virulence as a whole, we injected BALB/c mice with 1×10^6^ c.f.u. *B. pseudomallei* UKMH10 grown either in blood plasma or soil media via the intraperitoneal route and observed the mice over 10 days. As shown in Fig. 5c, the virulence of plasma and soil media-grown UKMH10 was not significantly different (logrank test; *P*-value=0.317311). All six mice infected with plasma-grown *B. pseudomallei* and 5/6 mice infected with soil media-grown *B. pseudomallei* died within 1 day post-infection.

## Discussion

*B. pseudomallei* is a soil-inhabiting pathogen that can cause a fatal infection in humans. As gene expression changes dynamically with different environments, a comparative profiling of *B. pseudomallei* transcriptional changes when grown in soil and human conditioned media is expected to provide insights into bacterial adaptation and virulence strategies essential for host invasion of soil-dwelling bacteria. In this study, we provide the first evidence of how gene expression of *B. pseudomallei* changes when grown in blood plasma relative to soil extract media. Conditioned growth of *B. pseudomallei* in human plasma to some extent models the gene expression profile during septicaemia, an acute form of melioidosis that always leads to patient death. This understanding could facilitate development of effective therapeutic strategies towards melioidosis.

An interesting insight from this study is that *B. pseudomallei* produces robust biofilm when grown in blood plasma. As human plasma contains soluble factors and globulins that could exert antibacterial activities, biofilm formation appears to be a strategy to ensure bacterial viability in blood. Biofilm is a bacterial aggregate embedded in exopolysaccharides (EPSs) that makes it more resistant to the host immune system or antimicrobial treatment compared to its planktonic counterpart. Franca and colleagues demonstrated that human whole blood or its blood components enhance biofilm formation in *Staphylococcus epidermis* [36–38], a well-known nosocomial pathogen. Here, we demonstrated that biofilm-forming ability is similarly enhanced in *B. pseudomallei* when grown in blood plasma. This may explain why *B. pseudomallei* can thrive in blood plasma and eventually cause septicaemic melioidosis. This speculation is further supported by the upregulation of the *arcDABC* gene cluster that is required for anaerobic survival and fitness within a biofilm during a mouse infection by *S. aureus *. The authors proposed that *arcDABC* is expressed to use arginine as the primary energy source under anaerobic conditions [39]

In tandem with the robust biofilm production during *B. pseudomallei* growth in plasma, type IV pili and flagella, the surface components that have important roles in bacterial attachment during the formation of biofilm were also positively regulated in plasma-cultured *B. pseudomallei* [33, 40]. These bacterial factors are also significant virulence factors for *B. pseudomallei *. Similarly, genes encoding the T3SS-3 were also induced in *B. pseudomallei *. A number of reports support the crucial role played in terms of virulence for *B. pseudomallei* T3SS-3 in various animal models [41, 42]. The positive regulation of genes within the T3SS-3 cluster might render the bacteria more virulent when present in a host-like niche (plasma) compared to bacteria that exist in the soil. These findings suggest that when *B. pseudomallei* shift to a host-like environment, they could display a more virulent phenotype. Although this was not reflected in the mouse infection model, we suggest that certain virulence factors or toxins that are expressed in low amounts when the bacteria are in soil-like conditions are stimulated in the shift to host-like conditions, resulting in the similar rate of mice killing.

Two-component systems (TCSs) are regulatory systems in bacteria that respond to diverse environmental cues by regulating various adaptation responses, including the formation of biofilm. They typically consist of a membrane-bound histidine kinase sensory protein and a response regulator protein. In *B. pseudomallei,* the *bfmR* gene has been well characterized as a TCS response gene responsible for adaptation to low-iron, thermal and pH-stress conditions [43, 44], as well as a positive regulator for the assembly of fimbriae and biofilm formation in *B. pseudomallei* [45]. In this study, we noted upregulation of several TCS genes in plasma-grown *B. pseudomallei *. We thus hypothesize that one of the TCS senses particular environmental stimuli in blood plasma, which in turn activates the transcriptional cascade, leading to the formation of biofilm in plasma-grown *B. pseudomallei *. Among the significantly upregulated TCS genes, *bpsl2367* was previously identified to also be highly expressed in *B. pseudomallei* strain UM6, a high biofilm producer [28]. Hence, the TCS protein BPSL2367 may have an important regulatory role in biofilm formation when the pathogen is within the infected host.

The present study demonstrated the positively regulated expression of several pathways involved in energy metabolism, such as carbohydrate and lipid metabolism, oxidative phosphorylation, nitrogen metabolism and phenylacetic acid (PAA) catabolism in plasma-grown *B. pseudomallei *. The differential expression of these energy metabolism genes in both plasma and soil media could also be an indication of the difference in nutrient composition in the media. Similarly, transcriptional analysis of *B. pseudomallei* RNA extracted from infected hamster organs showed an induction of expression for genes encoding enzymes required for oxidative phosphorylation and metabolism of alternative energy sources [44]. On the other hand, genes involved in glycolysis and oxidative phosphorylation were suppressed in intracellular *B. pseudomallei* during early infection of human macrophage cells [14]. Taken together, *B. pseudomallei* adapts it metabolism when in different environments depending on the availability of nutrients and energy sources. Additionally, activation of the PAA catabolism pathway was reported to be highly induced in *B. cenocepacia* grown under cystic fibrosis conditions [8, 9]. This metabolic pathway is responsible for degradation of aromatic compounds such as styrene, 2-phenylehtylamine and phenylalanine, with the end products serving as substrates for the tricarboxylic acid (TCA) cycle. Interestingly, there have been reports linking PAA catabolism with *B. cenocepacia* virulence. This pathway was first identified as essential for full virulence of *B. cenocepacia* in rats and *Caenorhabditis elegans* infection models [46, 47]. A follow-up study further attributed a relationship between virulence attenuation and dysfunctional PAA catabolism to reduced levels of quorum sensing, a key regulator for bacterial virulence [48]. To date, the role of PAA catabolism in *B. pseudomallei* has not been described. The known regulatory role of PAA in virulence of *B. cenocepacia* suggests a similar role for an activated PAA catabolism pathway in *B. pseudomallei* virulence.

A notable limitation of this study is that a study conducted *ex vivo* utilizing human plasma may not accurately reflect the conditions within a human body. The absence of several blood components, including immune cells (macrophage cells and T cells), in blood plasma limited a more accurate profiling of *B. pseudomallei* during a human infection. Nonetheless, we believe that the development of biofilm represents a major approach taken by *B. pseudomallei* to survive the human defence mechanism.

In summary, we report on the differential transcriptome profile of *B. pseudomallei* grown *ex vivo* in human blood plasma and in its natural growth environment, soil extract medium. This comparative study aims to provide insights into bacterial adaptations in a human host environment. In general, blood plasma triggers the expression of several known virulence factors of *B. pseudomallei *, including flagella, capsule, T3SS and biofilm formation, implying an enhanced virulence potential of plasma-grown *B. pseudomallei *. It is worth noting that biofilm-forming ability is robustly triggered in *B. pseudomallei* grown in blood plasma. As biofilm aggregates are more resistant to antibacterial treatments and host defence mechanisms, the formation of biofilm favours the establishment of infection and would aid disease progression. Thus, approaches that can diminish biofilm formation in *B. pseudomallei* will be of interest and applicable for melioidosis intervention.

## Supplementary Data

Supplementary material 1
