# Supplementary material for: Transcriptional landscape of Burkholderia pseudomallei cultured under environmental and clinical conditions
Source: Microb Genom. 2023 Apr 5;9(4):mgen000982. doi: 10.1099/mgen.0.000982 (PMC10210952; doi:10.1099/mgen.0.000982)
Supplement: Supplementary material 1 [file mgen-9-982-s001.pdf]

Table S1. Primer sequences used for quantitative RT-PCR analysis.

Gene	Functional category	Forward sequence (5' to 3')	Reverse sequence (5' to 3')
**Housekeeping gene**			
23s rRNA (1)		GTAGACCCGAAACCAGGTGA	CACCCCTATCCACAGCTCAT
**Upregulated genes**			
bpsl2308	Transport and binding	TGTGGGTGCCGTTCATCGTC	TCAGCCAGTTGTGCAGTCGG
bpsl1552	Cell envelope	GCCCGGAGAGGGCATCAATC	GTGCCGAGGATCAGCTCGAC
bpss0091	Biofilm formation	TCGATCGATCAGGGGCAGGT	CGAACCAGCGACATCTCCCG
bpss1545	Cellular process	GGACATGGAGGCGCTGATCC	CGGCTCTTGCGGATGTCGAT
bpsl1934	Cellular process	TCAAGATCGCGAAGGCGACG	GGATCGAAATGGCCCGCGTA
bpsl2366	Biosynthesis of cofactors	TGCAGCACACCCGTCGATTT	GCCGAGATACGCGTCGAACA
**Downregulated genes**			
bpsl0595	Hypothetical protein	GTCGAGCGGCACACAGTCAT	ACCTCGGTCTTGTTGCTGCC
bpss0213	Hypothetical protein	TGCTGAACGAAGGCTGGCAA	GATCGAATGCCAGCGGCTCT
bpsl3416	Amino acid transport and metabolism	CAGTCGTCGAAGGCGCAGAT	GTCAGCACGAGACCTTGCGT
bpss2037	Lipid transport and metabolism	ATCCGCATTCTCCGCACCTG	ATTCGCCGACGAACAGCCAA
bpss1734	Post-translational modification	ACCGAAGCGCAGGTGAAACA	ATGCACGAGCGACGTGTTGA
bpsl0592	Hypothetical protein	ACATCTCGCGGCAACTGGTC	GCGCAGACGGTCAGCTTGTA

References

1. Mima T, Schweizer HP, Xu ZQ. *In vitro* activity of cethromycin against *Burkholderia pseudomallei* and investigation of mechanism of resistance. *J Antimicrob Chemother*. 2011;66:73-78.

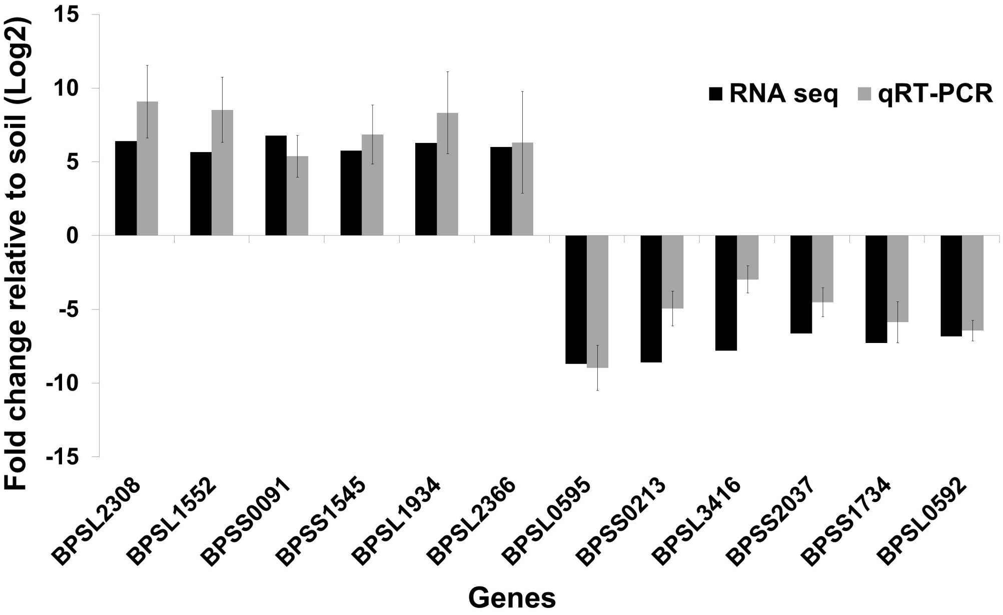

Fig. S1 Verification of sequencing data by qRT-PCR. The graph shows the relative expression ratios for qRT-PCR (grey bar) and RNA sequencing analysis (black bar). The vertical axis represents the $\log_2$ scale of the fold change. Values are averages of results from three independent biological replicates $\pm$ standard errors of the means.

Genes differentially expressed under the plasma versus soil conditions: Up regulated

gene_id	gene	locus	Soil	Plasma	log2 (fold_change)	test_stat	p_value	q_value	significant
BPSL0024	-	NC_006350.1:26222-26621	18.6988	746.197	5.31854	9.59356	5.00E-05	0.00324247	yes
BPSL0025	-	NC_006350.1:26689-27412	35.2528	1988.16	5.81755	6.43837	0.00015	0.00726516	yes
BPSL0030	*fliP*	NC_006350.1:30990-31752	18.9217	181.733	3.2637	3.8781	0.00285	0.0478054	yes
BPSL0106	-	NC_006350.1:117178-117748	16.6933	341.273	4.35358	5.27818	5.00E-05	0.00324247	yes
BPSL0269	*flgA*	NC_006350.1:280787-281552	11.6859	97.1323	3.05519	4.0772	0.00225	0.0411721	yes
BPSL0274	*flgF*	NC_006350.1:285777-286539	44.012	382.101	3.11798	3.44739	0.0028	0.0472736	yes
BPSL0275	*flgG*	NC_006350.1:286572-287361	47.8823	571.197	3.57643	3.25339	0.00195	0.0376377	yes
BPSL0276	*flgH*	NC_006350.1:287381-288104	21.4037	272.268	3.66909	3.65394	0.00155	0.0325081	yes
BPSL0277	*flgI*	NC_006350.1:288109-289303	30.361	265.561	3.12875	3.45486	0.0024	0.0426089	yes
BPSL0280	*flgK*	NC_006350.1:291418-293422	84.6923	960.499	3.50348	3.0342	0.00145	0.03117	yes
BPSL0281	*flgL*	NC_006350.1:293437-294670	134.294	1625.36	3.5973	3.10704	0.00255	0.0443077	yes
BPSL0328	-	NC_006350.1:350793-351753	83.5509	472.894	2.50079	2.92969	0.0023	0.04184	yes
BPSL0434	*glnB1*	NC_006350.1:473462-473801	45.3955	1266.49	4.80214	10.3781	5.00E-05	0.00324247	yes
BPSL0465	-	NC_006350.1:501739-503380	21.7065	154.678	2.83307	3.15991	0.00045	0.0148711	yes
BPSL0502	*cydA*	NC_006350.1:551744-553614	65.8252	1022.78	3.95771	3.47129	0.0012	0.027271	yes
BPSL0625	-	NC_006350.1:707730-709326	19.8999	155.94	2.97015	3.66996	0.00015	0.00726516	yes
BPSL0657	-	NC_006350.1:746085-747120	42.9518	319.415	2.89464	3.45557	0.0008	0.0213045	yes
BPSL0665	-	NC_006350.1:756144-756924	15.1505	108.452	2.83962	3.45992	0.0019	0.0369951	yes
BPSL0687	*glpK*	NC_006350.1:782877-784380	21.94	130.695	2.57457	2.6598	0.003	0.0495704	yes
BPSL0688	*glpD*	NC_006350.1:784485-786018	20.2263	253.162	3.64576	3.48171	0.0012	0.027271	yes
BPSL0711	-	NC_006350.1:814098-814437	0	61.438	inf	-nan	5.00E-05	0.00324247	yes
BPSL0735	-	NC_006350.1:849101-849665	31.8318	222.73	2.80676	4.13483	0.0024	0.0426089	yes
BPSL0816	*oprB*	NC_006350.1:947370-948915	8.01689	48.7665	2.60478	2.85676	0.00175	0.0349977	yes
BPSL1033	*hisJ*	NC_006350.1:1202830-1203607	55.4386	451.88	3.02698	3.73392	0.00045	0.0148711	yes
BPSL1263	-	NC_006350.1:1458135-1459878	23.2759	202.778	3.12299	3.46026	0.0006	0.0179525	yes
BPSL1339	-	NC_006350.1:1563719-1565207	34.6903	228.781	2.72136	3.33487	0.0015	0.0318473	yes
BPSL1406	-	NC_006350.1:1637454-1637784	53.7357	1298.69	4.59504	10.1639	0.0001	0.00541923	yes
BPSL1407	-	NC_006350.1:1638259-1638676	21.8166	296.838	3.76618	7.00801	0.00045	0.0148711	yes
BPSL1551	-	NC_006350.1:1800113-1801304	28.5184	561.405	4.29908	4.15371	0.00055	0.0171174	yes

BPSL1552	-	NC_006350.1:1801376-1802021	99.7937	5037.84	5.65771	5.54642	0.00045	0.0148711	yes
BPSL1607	*nosZ*	NC_006350.1:1860682-1862650	2.06065	32.3197	3.97124	2.98875	0.00045	0.0148711	yes
BPSL1631	-	NC_006350.1:1889770-1893145	1.79764	84.5777	5.5561	4.45685	5.00E-05	0.00324247	yes
BPSL1741	-	NC_006350.1:2053355-2053688	36.673	2693.03	6.19837	11.2226	5.00E-05	0.00324247	yes
BPSL1742	*arcD*	NC_006350.1:2053848-2055309	4.97384	726.922	7.19129	6.59752	5.00E-05	0.00324247	yes
BPSL1743	*arcA*	NC_006350.1:2055326-2056583	3.08151	1125.59	8.51283	7.11372	0.0001	0.00541923	yes
BPSL1744	*arcB*	NC_006350.1:2056647-2057658	5.23491	1604.21	8.25948	5.87647	5.00E-05	0.00324247	yes
BPSL1745	*arcC*	NC_006350.1:2057746-2058685	10.0371	1472.96	7.19723	5.80691	0.0001	0.00541923	yes
BPSL1746	-	NC_006350.1:2058927-2060168	5.00856	114.358	4.51302	4.83654	0.0001	0.00541923	yes
BPSL1751	-	NC_006350.1:2063490-2064606	4.04458	75.527	4.22293	4.02044	0.00045	0.0148711	yes
BPSL1768	*cobN*	NC_006350.1:2084040-2087886	13.2267	186.551	3.81805	3.81181	5.00E-05	0.00324247	yes
BPSL1769	*cobW*	NC_006350.1:2087952-2089026	19.9033	426.029	4.41987	5.00593	5.00E-05	0.00324247	yes
BPSL1770	*hoxN*	NC_006350.1:2089147-2090209	44.6711	324.715	2.86176	3.48089	0.00045	0.0148711	yes
BPSL1804	*amrA*	NC_006350.1:2150965-2152165	12.1568	115.099	3.24304	4.12432	0.0003	0.0117121	yes
BPSL1851	-	NC_006350.1:2204940-2206248	12.9201	247.106	4.25744	4.25209	0.0008	0.0213045	yes
BPSL2206	-	NC_006350.1:2648752-2648974	0	82.4292	inf	-nan	0.0021	0.0393566	yes
BPSL2299	-	NC_006350.1:2766718-2768488	53.3781	576.842	3.43386	3.16944	0.00035	0.0128852	yes
BPSL2300	*pdhB*	NC_006350.1:2768795-2770436	35.8795	496.155	3.78956	3.64782	0.0002	0.00888195	yes
BPSL2307	-	NC_006350.1:2782965-2784246	7.56799	393.673	5.70094	5.63501	5.00E-05	0.00324247	yes
BPSL2308	*narK*	NC_006350.1:2784289-2785675	3.41988	289.177	6.40187	5.50915	5.00E-05	0.00324247	yes
BPSL2309	*narG*	NC_006350.1:2785925-2791201	6.3671	484.346	6.24926	2.87942	0.00295	0.0489006	yes
BPSL2312	-	NC_006350.1:2791938-2792625	8.23467	352.479	5.41968	6.43587	5.00E-05	0.00324247	yes
BPSL2313	-	NC_006350.1:2792766-2794701	10.3161	455.573	5.46471	5.81644	5.00E-05	0.00324247	yes
BPSL2314	-	NC_006350.1:2794724-2795426	9.94157	183.849	4.2089	6.1901	5.00E-05	0.00324247	yes
BPSL2316	*glnG*	NC_006350.1:2796637-2798173	21.3481	722.473	5.08077	4.21637	0.0005	0.0162463	yes
BPSL2317	*glnL*	NC_006350.1:2798210-2799305	29.1935	462.736	3.98647	4.10606	0.0003	0.0117121	yes
BPSL2318	*glnA*	NC_006350.1:2799519-2800935	121.868	2219.81	4.18704	3.46221	0.0003	0.0117121	yes
BPSL2362	-	NC_006350.1:2854682-2855717	5.02271	112.391	4.48391	4.33203	0.00015	0.00726516	yes
BPSL2363	-	NC_006350.1:2855805-2857023	7.56627	184.325	4.60652	4.62241	0.0002	0.00888195	yes
BPSL2365	-	NC_006350.1:2857242-2859228	16.0416	700.53	5.44855	3.71749	0.00015	0.00726516	yes
BPSL2366	-	NC_006350.1:2859236-2860682	14.0075	910.107	6.02177	5.40531	5.00E-05	0.00324247	yes
BPSL2367	-	NC_006350.1:2860836-2862759	13.8129	257.097	4.21822	4.77014	5.00E-05	0.00324247	yes
BPSL2377	-	NC_006350.1:2872232-2872658	10.1091	263.334	4.70316	6.74958	0.00025	0.0105579	yes
BPSL2466	-	NC_006350.1:2972514-2973039	16.1682	99.9184	2.62759	3.91789	0.0028	0.0472736	yes

BPSL2501	-	NC_006350.1:3014503-3015298	16.1576	123.109	2.92966	3.33332	0.00205	0.0387003	yes
BPSL2545	*metE*	NC_006350.1:3071237-3073532	9.44358	77.3698	3.03437	3.44132	0.00165	0.0338272	yes
BPSL2608	-	NC_006350.1:3128726-3129845	14.3637	499.205	5.11914	4.96108	0.00025	0.0105579	yes
BPSL2610	-	NC_006350.1:3129945-3131731	11.5024	561.376	5.60896	2.7512	0.0028	0.0472736	yes
BPSL2611	*malE*	NC_006350.1:3131887-3133135	24.4357	621.501	4.66869	3.07135	0.0006	0.0179525	yes
BPSL2612	*zwf*	NC_006350.1:3133593-3135063	57.0128	397.347	2.80104	2.9506	0.00265	0.0456359	yes
BPSL2615	-	NC_006350.1:3138236-3139037	86.5435	1573.9	4.18477	3.87613	0.003	0.0495704	yes
BPSL2617	-	NC_006350.1:3139828-3140554	29.6884	337.534	3.50706	4.08655	0.00085	0.0219936	yes
BPSL2620	-	NC_006350.1:3142217-3143453	10.2272	79.1701	2.95254	3.90764	0.0005	0.0162463	yes
BPSL2646	*hemN*	NC_006350.1:3169252-3170647	21.4582	349.328	4.02498	4.34945	5.00E-05	0.00324247	yes
BPSL2651	-	NC_006350.1:3173858-3175166	33.2055	874.109	4.71832	3.60219	0.00205	0.0387003	yes
BPSL2652	-	NC_006350.1:3175260-3176883	9.72146	270.938	4.80064	4.06	0.0009	0.0229804	yes
BPSL2655	-	NC_006350.1:3178951-3179644	23.6974	1060.08	5.48331	5.53769	0.00015	0.00726516	yes
BPSL2662	*ureG*	NC_006350.1:3184783-3185434	12.4505	131.799	3.40406	4.53508	0.00015	0.00726516	yes
BPSL2669	-	NC_006350.1:3192942-3194100	13.2429	70.9671	2.42193	3.17395	0.00295	0.0489006	yes
BPSL2698	*groES*	NC_006350.1:3225166-3225460	692.393	10914.6	3.97853	12.9884	5.00E-05	0.00324247	yes
BPSL2776	-	NC_006350.1:3312990-3314187	0	12.2457	inf	-nan	5.00E-05	0.00324247	yes
BPSL2778	-	NC_006350.1:3315554-3317795	1.90446	12.4089	2.70393	2.90374	0.0022	0.0405926	yes
BPSL2782	-	NC_006350.1:3320834-3321881	6.44458	53.3827	3.05021	3.64861	0.00085	0.0219936	yes
BPSL2931	*eda*	NC_006350.1:3500447-3501077	74.5608	830.191	3.47695	4.63389	0.00015	0.00726516	yes
BPSL2932	*edd*	NC_006350.1:3501095-3502949	25.3862	283.608	3.48178	3.24596	0.0025	0.043832	yes
BPSL3197	*rplR*	NC_006350.1:3800837-3801203	175.196	2651.13	3.91956	6.13918	0.00285	0.0478054	yes
BPSL3208	*rplV*	NC_006350.1:3805746-3806076	181.017	4017	4.47192	9.23946	0.00095	0.0237485	yes
BPSL3209	*rpsS*	NC_006350.1:3806088-3806364	151.787	2121.04	3.80465	9.78467	0.0015	0.0318473	yes
BPSL3232	*paaC*	NC_006350.1:3839967-3840771	17.8857	285.419	3.9962	4.33215	0.0008	0.0213045	yes
BPSL3233	*paaD*	NC_006350.1:3840806-3841427	4.23556	90.6275	4.41932	5.02914	0.0024	0.0426089	yes
BPSL3234	*paaE*	NC_006350.1:3841428-3842517	13.5574	205.842	3.92439	3.6628	0.00105	0.0253488	yes
BPSL3237	-	NC_006350.1:3844298-3844985	118.033	5441.21	5.52666	6.39701	5.00E-05	0.00324247	yes
BPSL3254A	-	NC_006350.1:3867515-3867743	312.652	6025.06	4.26835	46.765	0.0001	0.00541923	yes
BPSL3300	*cheY*	NC_006350.1:3918812-3919241	150.423	919.899	2.61245	4.52866	0.00265	0.0456359	yes
BPSL3320	*fliD*	NC_006350.1:3938990-3940511	92.44	953.486	3.36662	2.93871	0.0011	0.0261486	yes
BPSL3321	-	NC_006350.1:3940524-3940839	56.829	377.749	2.73273	7.3236	0.00195	0.0376377	yes
BPSL3330	-	NC_006350.1:3949406-3950144	46.6963	386.52	3.04916	3.14663	0.0018	0.0357211	yes
BPSL3331	-	NC_006350.1:3950468-3950867	42.1195	276.898	2.7168	5.05868	0.00155	0.0325081	yes

BPSL3336	-	NC_006350.1:3955730-3956408	108.269	968.14	3.16059	3.81627	0.00175	0.0349977	yes
BPSS0031	-	NC_006351.1:30313-31069	345.721	4047.99	3.54952	4.22462	0.0014	0.0303053	yes
BPSS0091	-	NC_006351.1:110181-110727	6.45187	702.374	6.76638	7.41027	0.0002	0.00888195	yes
BPSS0093	-	NC_006351.1:111752-114951	1.49778	11.2379	2.90747	2.67846	0.00225	0.0411721	yes
BPSS0098	-	NC_006351.1:118301-119801	14.3315	251.337	4.13236	3.78138	5.00E-05	0.00324247	yes
BPSS0099	-	NC_006351.1:120000-120483	15.6176	1952.19	6.96578	9.95157	5.00E-05	0.00324247	yes
BPSS0101	-	NC_006351.1:121158-123806	3.96981	44.6008	3.48993	2.99009	0.0023	0.04184	yes
BPSS0103	-	NC_006351.1:123820-127729	3.80037	39.7657	3.38731	3.47167	0.0001	0.00541923	yes
BPSS0105	-	NC_006351.1:128552-131252	3.03818	23.2264	2.93449	2.98922	0.0004	0.0139317	yes
BPSS0123	-	NC_006351.1:157967-158624	24.3325	997.19	5.35691	5.04603	0.0001	0.00541923	yes
BPSS0144	-	NC_006351.1:186667-189283	17.4027	214.172	3.62138	3.01037	0.0003	0.0117121	yes
BPSS0220	-	NC_006351.1:296381-297662	28.361	257.872	3.18468	3.61344	0.00095	0.0237485	yes
BPSS0221A	-	NC_006351.1:297813-298842	85.7446	5786.61	6.07653	4.51766	0.0014	0.0303053	yes
BPSS0228	-	NC_006351.1:311610-311874	149.458	3426.19	4.5188	20.0834	0.00015	0.00726516	yes
BPSS0234	-	NC_006351.1:318008-319016	3.18387	406.918	6.99781	7.78932	0.0001	0.00541923	yes
BPSS0235	-	NC_006351.1:319051-320464	8.39457	535.001	5.99394	5.82618	5.00E-05	0.00324247	yes
BPSS0276	*aer*	NC_006351.1:367569-369114	6.328	269.315	5.4114	5.86113	5.00E-05	0.00324247	yes
BPSS0277	-	NC_006351.1:369206-370223	11.8473	128.435	3.43841	4.03317	0.00035	0.0128852	yes
BPSS0481	-	NC_006351.1:652894-654520	6.03658	38.4409	2.67084	3.25887	0.00125	0.0281595	yes
BPSS0547	*glyA*	NC_006351.1:747750-749040	11.3979	114.16	3.32422	3.09209	0.00265	0.0456359	yes
BPSS0548	-	NC_006351.1:749112-750084	17.5064	319.632	4.19045	3.92346	0.0007	0.019907	yes
BPSS0549	-	NC_006351.1:750234-750768	6.26964	113.147	4.17367	5.3327	0.00095	0.0237485	yes
BPSS0550	-	NC_006351.1:750828-752892	5.29939	78.8515	3.89524	3.2689	0.00095	0.0237485	yes
BPSS0551	-	NC_006351.1:752894-754820	8.4886	124.333	3.87254	3.28927	0.002	0.0381749	yes
BPSS0552	-	NC_006351.1:754824-756779	4.59282	103.633	4.49596	4.07279	0.00045	0.0148711	yes
BPSS0554	-	NC_006351.1:756803-758072	8.84655	185.843	4.39282	3.94821	0.0004	0.0139317	yes
BPSS0556	-	NC_006351.1:759347-760211	17.234	287.752	4.06149	4.07073	0.00115	0.0268431	yes
BPSS0558	-	NC_006351.1:762133-763009	27.355	235.442	3.10549	3.61247	0.00115	0.0268431	yes
BPSS0689	-	NC_006351.1:930918-931698	14.6526	119.766	3.03099	3.67055	0.0025	0.043832	yes
BPSS0721	-	NC_006351.1:971274-972036	5.69475	82.4135	3.85518	4.49177	0.00075	0.0206837	yes
BPSS0839	-	NC_006351.1:1124883-1125723	16.5141	509.514	4.94735	5.04321	0.00015	0.00726516	yes
BPSS0840	-	NC_006351.1:1125972-1127013	6.57764	193.254	4.87679	4.95792	0.0001	0.00541923	yes
BPSS1038	-	NC_006351.1:1414650-1414923	171.904	4606.08	4.74386	22.2241	5.00E-05	0.00324247	yes
BPSS1042	*czcB*	NC_006351.1:1420383-1421853	3.743	42.6948	3.5118	4.02315	0.0002	0.00888195	yes

BPSS1043	*czcC*	NC_006351.1:1421863-1423222	2.76101	28.2158	3.35324	3.88571	0.0009	0.0229804	yes
BPSS1100	-	NC_006351.1:1476379-1478893	2.61527	22.7738	3.12234	3.54993	0.00115	0.0268431	yes
BPSS1101	-	NC_006351.1:1478990-1479470	10.7652	500.65	5.53936	8.14964	0.0001	0.00541923	yes
BPSS1112	-	NC_006351.1:1491118-1492609	9.26008	295.79	4.99741	5.24251	5.00E-05	0.00324247	yes
BPSS1140	-	NC_006351.1:1526485-1527448	107.893	1278.22	3.56646	4.04953	0.0015	0.0318473	yes
BPSS1239	-	NC_006351.1:1673067-1674069	9.62825	73.5538	2.93345	3.47931	0.00105	0.0253488	yes
BPSS1240	*ftsI*	NC_006351.1:1674423-1676136	5.60899	44.4835	2.98746	3.40775	0.00075	0.0206837	yes
BPSS1241	-	NC_006351.1:1676704-1680961	5.82673	202.428	5.11858	3.60638	0.0012	0.027271	yes
BPSS1242	-	NC_006351.1:1681004-1681355	16.7402	1319.77	6.30083	11.7902	0.00015	0.00726516	yes
BPSS1243	*nirB*	NC_006351.1:1681392-1683978	16.0078	675.161	5.39838	3.62542	0.0016	0.0331087	yes
BPSS1244	-	NC_006351.1:1683991-1685317	8.36271	147.279	4.13844	3.92616	0.0011	0.0261486	yes
BPSS1245	-	NC_006351.1:1686327-1687152	4.52287	151.508	5.06601	5.15086	0.00015	0.00726516	yes
BPSS1353	*betI*	NC_006351.1:1848476-1849064	9.85162	129.156	3.71261	5.2697	0.0003	0.0117121	yes
BPSS1354	*betB*	NC_006351.1:1849106-1850576	18.5376	623.178	5.07112	4.83953	5.00E-05	0.00324247	yes
BPSS1355	*betA*	NC_006351.1:1850595-1852293	26.514	381.424	3.84657	3.52647	0.00055	0.0171174	yes
BPSS1369	*soxB*	NC_006351.1:1873102-1874347	3.12144	53.0059	4.08587	3.71161	0.00065	0.0191164	yes
BPSS1423	-	NC_006351.1:1937182-1938133	12.5717	138.512	3.46177	3.40588	0.0029	0.0484866	yes
BPSS1424	-	NC_006351.1:1938233-1939232	4.57912	48.731	3.4117	3.53682	0.0025	0.043832	yes
BPSS1467	-	NC_006351.1:2001095-2002322	14.7871	180.948	3.61316	3.45889	0.00295	0.0489006	yes
BPSS1468	-	NC_006351.1:2002376-2003396	11.7987	190.513	4.01319	4.03484	0.001	0.0247193	yes
BPSS1534	*bsaZ*	NC_006351.1:2088081-2089317	5.66845	83.1711	3.87506	4.46556	5.00E-05	0.00324247	yes
BPSS1535	*bsaY*	NC_006351.1:2089320-2090076	5.03283	73.853	3.87521	4.91679	0.00075	0.0206837	yes
BPSS1543	*bsaQ*	NC_006351.1:2095477-2097550	4.08162	116.264	4.83212	4.79512	5.00E-05	0.00324247	yes
BPSS1545	*bsaO*	NC_006351.1:2097585-2100551	5.38728	290.707	5.75386	3.93663	5.00E-05	0.00324247	yes
BPSS1546	*bsaN*	NC_006351.1:2100564-2101206	6.62593	453.924	6.09818	6.89634	0.0001	0.00541923	yes
BPSS1550	-	NC_006351.1:2103635-2105918	5.59784	254.244	5.5052	5.28768	0.00135	0.0298483	yes
BPSS1582	*bcsB*	NC_006351.1:2151583-2153980	0.763976	17.6274	4.52815	4.47747	0.0028	0.0472736	yes
BPSS1733	-	NC_006351.1:2380631-2380766	0	10594.9	inf	-nan	0.0003	0.0117121	yes
BPSS1748	-	NC_006351.1:2397871-2398204	245.525	3548.45	3.85325	9.23873	0.0006	0.0179525	yes
BPSS1783	-	NC_006351.1:2442025-2442184	14050.2	90493.6	2.68722	647.136	0.0015	0.0318473	yes
BPSS1918	-	NC_006351.1:2599461-2600675	11.8561	121.146	3.35304	4.12317	0.00175	0.0349977	yes
BPSS1920	-	NC_006351.1:2601412-2601841	37.7819	848.282	4.48878	7.54066	0.0001	0.00541923	yes
BPSS1921	-	NC_006351.1:2602226-2602550	21.9098	1091.07	5.63802	11.9278	0.0001	0.00541923	yes
BPSS1932	-	NC_006351.1:2615276-2616661	9.09951	579.393	5.99261	4.83985	0.0001	0.00541923	yes

BPSS1934	-	NC_006351.1:2616693-2617164	21.5142	1675.37	6.28304	9.69174	5.00E-05	0.00324247	yes
BPSS1939	-	NC_006351.1:2624359-2625484	3.40092	164.646	5.5973	5.577	5.00E-05	0.00324247	yes
BPSS1944	*adhA*	NC_006351.1:2631077-2632103	7.05899	275.265	5.28521	5.38486	5.00E-05	0.00324247	yes
BPSS1949	-	NC_006351.1:2635909-2637152	6.54352	121.545	4.21528	3.83202	0.0002	0.00888195	yes
BPSS1953	*atpD*	NC_006351.1:2637650-2639631	2.25939	339.963	7.2333	6.78982	0.0003	0.0117121	yes
BPSS1954	-	NC_006351.1:2640072-2641866	7.43342	192.039	4.69123	4.04254	0.0002	0.00888195	yes
BPSS1955	-	NC_006351.1:2641876-2644455	2.31922	141.512	5.93114	4.41259	0.002	0.0381749	yes
BPSS1958	-	NC_006351.1:2646620-2647268	9.84042	203.81	4.37236	5.95897	5.00E-05	0.00324247	yes
BPSS1996	-	NC_006351.1:2700918-2701311	259.068	39275.5	7.24416	9.79733	5.00E-05	0.00324247	yes
BPSS1997	*oxa*	NC_006351.1:2701463-2702273	33.6993	1052.81	4.96538	5.83607	5.00E-05	0.00324247	yes
BPSS2162	-	NC_006351.1:2924881-2926009	13.0867	71.8843	2.45758	3.54797	0.00155	0.0325081	yes
BPSS2282	-	NC_006351.1:3068153-3069995	2.4408	19.9126	3.02825	3.38728	0.00285	0.0478054	yes
BPSS2284	-	NC_006351.1:3072405-3075266	9.12931	387.545	5.40771	5.17751	0.00065	0.0191164	yes
BPSS2287	-	NC_006351.1:3076640-3077294	46.4573	730.458	3.97482	5.0354	0.00085	0.0219936	yes
BPSS2288	-	NC_006351.1:3077354-3077789	63.0735	2057.76	5.0279	6.60244	0.0002	0.00888195	yes
BPSS2302	-	NC_006351.1:3097925-3098816	2.99324	38.0878	3.66955	4.44082	0.0024	0.0426089	yes

Genes differentially expressed under the plasma versus soil conditions: Down regulated

gene_id	gene	locus	Soil	Plasma	log2 (fold_change)	test_stat	p_value	q_value	significant
BPSL0050	-	NC_006350.1:54122-55382	34.5306	5.88408	-2.55299	-2.90468	0.0018	0.0357211	yes
BPSL0093	-	NC_006350.1:105282-106440	117.36	23.001	-2.35117	-2.73394	0.0029	0.0484866	yes
BPSL0095	-	NC_006350.1:107004-107316	2265.57	335.628	-2.75494	-8.58575	0.00055	0.0171174	yes
BPSL0097	-	NC_006350.1:108382-108727	33004.3	214.371	-7.2664	-13.8264	5.00E-05	0.00324247	yes
BPSL0216	-	NC_006350.1:225079-225265	606.829	0	-inf	-nan	0.00255	0.0443077	yes
BPSL0296	-	NC_006350.1:312293-312518	5288.78	728.666	-2.8596	-38.7509	0.0003	0.0117121	yes
BPSL0311	-	NC_006350.1:331092-331539	1405.39	119.212	-3.55937	-6.38271	5.00E-05	0.00324247	yes
BPSL0348	-	NC_006350.1:371476-372250	283.241	24.5537	-3.52801	-3.80715	5.00E-05	0.00324247	yes
BPSL0349	-	NC_006350.1:372395-372953	6778.32	62.4508	-6.76206	-6.37443	5.00E-05	0.00324247	yes
BPSL0350	-	NC_006350.1:373422-374079	100.78	10.8601	-3.2141	-4.68005	0.0002	0.00888195	yes
BPSL0495	-	NC_006350.1:538359-539124	61.3454	9.37162	-2.71058	-4.2917	0.0025	0.043832	yes
BPSL0499	-	NC_006350.1:544078-545851	603.029	30.1385	-4.32255	-4.50375	5.00E-05	0.00324247	yes
BPSL0500	-	NC_006350.1:546568-549070	111.555	13.4763	-3.04926	-3.20341	0.0003	0.0117121	yes
BPSL0590	-	NC_006350.1:654015-660111	26.6783	1.40634	-4.24564	-4.27595	5.00E-05	0.00324247	yes
BPSL0591	-	NC_006350.1:660132-663417	47.7517	7.5352	-2.66383	-2.63532	0.00075	0.0206837	yes
BPSL0592	-	NC_006350.1:664601-664985	1741.88	15.3067	-6.83034	-13.1608	5.00E-05	0.00324247	yes
BPSL0593	-	NC_006350.1:665002-665440	1127.5	17.5449	-6.00593	-10.6967	5.00E-05	0.00324247	yes
BPSL0595	-	NC_006350.1:666527-666893	14236.9	34.3115	-8.69672	-17.0714	5.00E-05	0.00324247	yes
BPSL0596	-	NC_006350.1:667140-668148	734.144	26.1188	-4.8129	-5.44433	5.00E-05	0.00324247	yes
BPSL0597	-	NC_006350.1:668158-672597	195.656	7.36309	-4.73186	-3.38313	0.00145	0.03117	yes
BPSL0599	-	NC_006350.1:672598-672961	8411.15	143.829	-5.86987	-11.5517	5.00E-05	0.00324247	yes
BPSL0600	-	NC_006350.1:673052-675353	188.605	7.36025	-4.67947	-5.15805	5.00E-05	0.00324247	yes
BPSL0601	-	NC_006350.1:675422-676199	211.534	9.05227	-4.54647	-5.77342	5.00E-05	0.00324247	yes
BPSL0690	-	NC_006350.1:786623-786833	5954.99	554.275	-3.42543	-79.8899	0.0004	0.0139317	yes
BPSL0706	-	NC_006350.1:806552-808304	78.8179	11.4288	-2.78584	-2.93668	0.0009	0.0229804	yes
BPSL0718	-	NC_006350.1:821673-822381	131.729	15.0227	-3.13235	-4.83583	0.0004	0.0139317	yes
BPSL0773	-	NC_006350.1:898929-900600	414.854	5.32619	-6.28335	-6.35999	5.00E-05	0.00324247	yes
BPSL0775	-	NC_006350.1:902390-903134	417.283	57.9164	-2.84898	-4.04185	0.0016	0.0331087	yes
BPSL1007	-	NC_006350.1:1171460-1173761	101.04	9.88827	-3.35307	-3.74381	5.00E-05	0.00324247	yes

BPSL1008	-	NC_006350.1:1174515-1175226	885.272	41.2453	-4.42382	-5.78483	5.00E-05	0.00324247	yes
BPSL1009	-	NC_006350.1:1175233-1175752	2667.94	366.29	-2.86467	-4.2268	0.00195	0.0376377	yes
BPSL1059	-	NC_006350.1:1227013-1227298	8109.69	284.014	-4.83561	-16.8505	5.00E-05	0.00324247	yes
BPSL1060	-	NC_006350.1:1227308-1227826	1550.12	93.6759	-4.04856	-11.5792	0.00025	0.0105579	yes
BPSL1063	-	NC_006350.1:1228625-1228970	253.73	37.4594	-2.75989	-7.60102	0.00245	0.0433477	yes
BPSL1078	-	NC_006350.1:1246812-1247838	121.886	20.204	-2.59282	-3.16891	0.0025	0.043832	yes
BPSL1079	-	NC_006350.1:1248217-1249411	2302.8	88.4582	-4.70225	-5.68744	5.00E-05	0.00324247	yes
BPSL1093	-	NC_006350.1:1262587-1264374	163.284	15.8148	-3.36804	-3.14708	0.0002	0.00888195	yes
BPSL1306	-	NC_006350.1:1524127-1525492	429.235	35.2126	-3.60761	-3.94823	5.00E-05	0.00324247	yes
BPSL1308	-	NC_006350.1:1526859-1527657	68.5661	5.5648	-3.62309	-4.45796	0.002	0.0381749	yes
BPSL1348	-	NC_006350.1:1573866-1574889	1018.75	104.819	-3.28084	-3.86324	0.00025	0.0105579	yes
BPSL1374	-	NC_006350.1:1604280-1604742	669.926	91.6087	-2.87045	-5.23793	0.0004	0.0139317	yes
BPSL1376	-	NC_006350.1:1606062-1606326	1307.52	177.111	-2.88411	-16.6487	0.0004	0.0139317	yes
BPSL1383	*uppP*	NC_006350.1:1613302-1614133	597.582	48.2685	-3.62998	-4.30359	0.0001	0.00541923	yes
BPSL1390	-	NC_006350.1:1617922-1618318	2034.66	25.4018	-6.32371	-11.133	5.00E-05	0.00324247	yes
BPSL1393	-	NC_006350.1:1619143-1619650	1030.56	26.8828	-5.26061	-7.17626	5.00E-05	0.00324247	yes
BPSL1427	-	NC_006350.1:1664741-1665281	380.68	59.4918	-2.67782	-4.15516	0.001	0.0247193	yes
BPSL1549	-	NC_006350.1:1797527-1798163	1216.27	111.587	-3.44622	-4.01806	0.0003	0.0117121	yes
BPSL1575	-	NC_006350.1:1830637-1831627	350.393	46.2347	-2.92193	-3.83276	0.0006	0.0179525	yes
BPSL1616	-	NC_006350.1:1872875-1874255	25.2943	3.41741	-2.88783	-3.61779	0.0019	0.0369951	yes
BPSL1617	-	NC_006350.1:1874295-1875738	63.0821	7.40819	-3.09004	-3.35972	0.00035	0.0128852	yes
BPSL1618	-	NC_006350.1:1875875-1876574	148.289	6.74624	-4.45818	-5.16936	0.0002	0.00888195	yes
BPSL1649	-	NC_006350.1:1911564-1912596	103.184	13.2661	-2.9594	-3.46047	0.00025	0.0105579	yes
BPSL1655	-	NC_006350.1:1917833-1918952	45.0721	5.36474	-3.07065	-3.21952	0.00045	0.0148711	yes
BPSL1658a	-	NC_006350.1:1921336-1921723	662.01	20.2044	-5.03411	-8.9223	5.00E-05	0.00324247	yes
BPSL1659	-	NC_006350.1:1922209-1923109	448.747	13.6734	-5.03645	-5.80277	5.00E-05	0.00324247	yes
BPSL1660	-	NC_006350.1:1923237-1925037	345.051	20.3661	-4.08257	-3.84567	5.00E-05	0.00324247	yes
BPSL1661	-	NC_006350.1:1925147-1934837	329.401	24.0323	-3.7768	-2.54703	0.00135	0.0298483	yes
BPSL1662	-	NC_006350.1:1934932-1936314	641.399	25.8582	-4.63253	-4.96007	0.0006	0.0179525	yes
BPSL1668	-	NC_006350.1:1946219-1947071	202.514	33.9377	-2.57706	-3.24363	0.00125	0.0281595	yes
BPSL1669	-	NC_006350.1:1948083-1948797	240.884	25.7298	-3.22683	-3.891	0.0002	0.00888195	yes
BPSL1710	-	NC_006350.1:1994316-1996689	50.8716	6.53638	-2.96029	-3.26025	0.0007	0.019907	yes
BPSL1711	-	NC_006350.1:1996807-1998529	332	25.5412	-3.70029	-3.77967	5.00E-05	0.00324247	yes
BPSL1712	-	NC_006350.1:1998619-2018013	106.304	9.9319	-3.41999	-2.98568	0.00105	0.0253488	yes

BPSL1714	-	NC_006350.1:2018069-2019230	135.504	17.716	-2.93521	-3.7407	0.0004	0.0139317	yes
BPSL1715	-	NC_006350.1:2019272-2021957	213.039	9.98908	-4.41462	-4.4441	5.00E-05	0.00324247	yes
BPSL1716	-	NC_006350.1:2022009-2023125	237.273	13.3907	-4.14724	-4.62025	5.00E-05	0.00324247	yes
BPSL1718	-	NC_006350.1:2023133-2025004	268.968	6.38586	-5.39641	-4.95275	5.00E-05	0.00324247	yes
BPSL1721	-	NC_006350.1:2025057-2028583	743.219	25.396	-4.87111	-2.69442	0.0012	0.027271	yes
BPSL1722	-	NC_006350.1:2028623-2029442	478.941	15.0776	-4.98937	-5.6264	5.00E-05	0.00324247	yes
BPSL1723	-	NC_006350.1:2029497-2030598	997.897	24.8573	-5.32715	-5.41981	5.00E-05	0.00324247	yes
BPSL1724	-	NC_006350.1:2030607-2031618	1190.67	23.2569	-5.67797	-6.47594	5.00E-05	0.00324247	yes
BPSL1725	-	NC_006350.1:2031679-2032775	3651.33	43.6356	-6.38677	-5.29376	5.00E-05	0.00324247	yes
BPSL1727	-	NC_006350.1:2033809-2035639	96.682	12.0632	-3.00263	-3.22266	0.00065	0.0191164	yes
BPSL1818	-	NC_006350.1:2165511-2166831	58.9114	2.11436	-4.80026	-5.16191	5.00E-05	0.00324247	yes
BPSL1834	-	NC_006350.1:2187590-2188553	1138.06	95.7497	-3.57116	-3.73845	0.00015	0.00726516	yes
BPSL1876	-	NC_006350.1:2232807-2234328	75.5407	7.77643	-3.28007	-3.54278	0.00015	0.00726516	yes
BPSL1877	-	NC_006350.1:2234634-2236161	66.2574	6.30074	-3.39449	-4.08104	5.00E-05	0.00324247	yes
BPSL1878	-	NC_006350.1:2236552-2238265	234.392	32.531	-2.84904	-3.50835	0.0006	0.0179525	yes
BPSL1879	-	NC_006350.1:2238311-2238938	629.482	85.2293	-2.88474	-3.99486	0.00065	0.0191164	yes
BPSL1881	-	NC_006350.1:2240051-2241272	837.842	40.1227	-4.38419	-5.05568	5.00E-05	0.00324247	yes
BPSL1885	-	NC_006350.1:2245608-2245881	1539.45	185.477	-3.0531	-14.3416	0.0007	0.019907	yes
BPSL1887	-	NC_006350.1:2247790-2249182	477.645	66.7973	-2.83808	-3.06123	0.00105	0.0253488	yes
BPSL1888	-	NC_006350.1:2249198-2250905	301.9	24.0891	-3.64762	-3.55472	0.00035	0.0128852	yes
BPSL1889	-	NC_006350.1:2251014-2251410	554.617	74.8139	-2.89011	-6.20475	0.0008	0.0213045	yes
BPSL1890	-	NC_006350.1:2251431-2252397	278.214	25.6082	-3.44152	-4.32688	0.0001	0.00541923	yes
BPSL1893	-	NC_006350.1:2252403-2255769	851.731	53.7584	-3.98584	-2.59197	0.0015	0.0318473	yes
BPSL1894	-	NC_006350.1:2255772-2257011	246.64	15.3516	-4.00595	-5.12338	5.00E-05	0.00324247	yes
BPSL1895	-	NC_006350.1:2257077-2258403	824.303	41.9443	-4.29663	-4.7705	5.00E-05	0.00324247	yes
BPSL1896	-	NC_006350.1:2258466-2259366	398.851	15.0998	-4.72325	-6.09253	5.00E-05	0.00324247	yes
BPSL1898	-	NC_006350.1:2259445-2260410	771.495	47.4916	-4.02191	-2.68526	0.0008	0.0213045	yes
BPSL1899	-	NC_006350.1:2260492-2260663	36343.4	2472.25	-3.87779	-465.131	0.00015	0.00726516	yes
BPSL1900	-	NC_006350.1:2260770-2260908	15228.5	0	-inf	-nan	5.00E-05	0.00324247	yes
BPSL1901	-	NC_006350.1:2261327-2262917	18.0023	1.90651	-3.23917	-3.08611	0.00245	0.0433477	yes
BPSL1903	-	NC_006350.1:2264382-2266062	149.704	14.0477	-3.4137	-3.57977	5.00E-05	0.00324247	yes
BPSL1935	-	NC_006350.1:2303058-2304126	66.1965	9.76612	-2.7609	-3.2385	0.0008	0.0213045	yes
BPSL1958	-	NC_006350.1:2331167-2332241	1544.62	121.304	-3.67056	-3.72569	0.0002	0.00888195	yes
BPSL1991	-	NC_006350.1:2373227-2374277	73.5039	14.6593	-2.326	-2.70977	0.00295	0.0489006	yes

BPSL2008	-	NC_006350.1:2394124-2395090	188.665	30.5473	-2.62671	-3.59701	0.00085	0.0219936	yes
BPSL2073	*phaZ*	NC_006350.1:2480504-2481983	1313.66	14.7501	-6.47672	-5.81713	5.00E-05	0.00324247	yes
BPSL2082	-	NC_006350.1:2501375-2501753	637.37	65.6837	-3.27852	-6.82891	0.00015	0.00726516	yes
BPSL2110	-	NC_006350.1:2535551-2535761	748.265	0	-inf	-nan	5.00E-05	0.00324247	yes
BPSL2189	-	NC_006350.1:2628232-2628667	504.015	70.855	-2.83053	-5.76658	0.0007	0.019907	yes
BPSL2290	-	NC_006350.1:2757471-2758011	699.162	100.572	-2.7974	-4.24209	0.0014	0.0303053	yes
BPSL2347	-	NC_006350.1:2837795-2838521	164.518	3.87199	-5.40902	-6.28806	0.00115	0.0268431	yes
BPSL2350	-	NC_006350.1:2841999-2843166	298.347	25.5421	-3.54604	-4.16167	5.00E-05	0.00324247	yes
BPSL2557	*cspA*	NC_006350.1:3086218-3086422	83966.8	8988.09	-3.22373	-107.283	0.0002	0.00888195	yes
BPSL2558	-	NC_006350.1:3086824-3087088	3306.11	201.161	-4.03871	-21.3529	5.00E-05	0.00324247	yes
BPSL2589	-	NC_006350.1:3108887-3109382	239.6	35.7505	-2.74459	-4.65575	0.0012	0.027271	yes
BPSL2599	-	NC_006350.1:3118610-3118883	4561.1	357.569	-3.67309	-15.0139	5.00E-05	0.00324247	yes
BPSL2634	-	NC_006350.1:3158310-3158604	353.405	47.1729	-2.90529	-11.3184	0.00155	0.0325081	yes
BPSL2639	-	NC_006350.1:3162210-3163431	1109.65	57.4298	-4.27217	-4.62937	0.0001	0.00541923	yes
BPSL2703	-	NC_006350.1:3232493-3233009	16702.7	488.883	-5.09445	-4.73376	0.0016	0.0331087	yes
BPSL2706	-	NC_006350.1:3235339-3236938	370.707	40.1019	-3.20854	-3.12941	0.00025	0.0105579	yes
BPSL3036	-	NC_006350.1:3617870-3619031	2534.7	182.782	-3.79362	-3.36448	0.0012	0.027271	yes
BPSL3108	-	NC_006350.1:3713278-3713950	726.087	96.7553	-2.90773	-4.08247	0.0008	0.0213045	yes
BPSL3109	-	NC_006350.1:3714324-3714939	179.153	19.6396	-3.18936	-4.13525	0.0003	0.0117121	yes
BPSL3160	-	NC_006350.1:3764661-3765375	2600.94	326.103	-2.99563	-3.68353	0.0028	0.0472736	yes
BPSL3247	-	NC_006350.1:3858607-3860011	136.294	13.9077	-3.29277	-3.42271	0.0002	0.00888195	yes
BPSL3248	-	NC_006350.1:3860136-3861387	76.295	8.85289	-3.10737	-3.04726	0.00015	0.00726516	yes
BPSL3313	-	NC_006350.1:3932434-3932731	16307.9	1443.53	-3.4979	-9.05243	0.0008	0.0213045	yes
BPSL3314	*aqpZ*	NC_006350.1:3933036-3933741	1355.04	152.416	-3.15225	-4.37465	0.0002	0.00888195	yes
BPSL3339	-	NC_006350.1:3960413-3962075	342.006	12.9166	-4.72672	-4.30408	5.00E-05	0.00324247	yes
BPSL3340	-	NC_006350.1:3962263-3962965	5389.8	56.0732	-6.58678	-5.91341	5.00E-05	0.00324247	yes
BPSL3354	-	NC_006350.1:3976224-3977124	373.668	35.6108	-3.39137	-3.98321	0.0003	0.0117121	yes
BPSL3412	-	NC_006350.1:4051534-4052692	223.386	38.5999	-2.53287	-3.06984	0.0018	0.0357211	yes
BPSL3414	-	NC_006350.1:4054657-4056516	1763.33	56.4982	-4.96396	-4.94841	0.00055	0.0171174	yes
BPSL3416	-	NC_006350.1:4056655-4057852	3567.73	16.093	-7.79243	-6.82064	5.00E-05	0.00324247	yes
BPSL3418	-	NC_006350.1:4057919-4059451	1752.25	18.2013	-6.58902	-4.75777	5.00E-05	0.00324247	yes
BPSLr01	-	NC_006350.1:1431767-1433301	167.024	26.4469	-2.65888	-2.30667	0.00195	0.0376377	yes
BPSS0024	-	NC_006351.1:23466-23667	30144.2	901.206	-5.06388	-171.92	5.00E-05	0.00324247	yes
BPSS0027	-	NC_006351.1:23840-27462	690.704	24.7746	-4.80114	-3.12865	0.00055	0.0171174	yes

BPSS0028	-	NC_006351.1:27475-28465	335.665	14.3687	-4.54602	-5.59248	5.00E-05	0.00324247	yes
BPSS0049	-	NC_006351.1:48430-48679	1349.27	182.589	-2.8855	-19.9597	0.0014	0.0303053	yes
BPSS0169	-	NC_006351.1:226855-228202	24.9908	2.94252	-3.08627	-3.24038	0.0019	0.0369951	yes
BPSS0171	-	NC_006351.1:228833-229319	224.693	6.50076	-5.1112	-7.41506	0.00075	0.0206837	yes
BPSS0172	-	NC_006351.1:229435-230935	95.9463	5.02255	-4.25573	-4.33178	5.00E-05	0.00324247	yes
BPSS0173	-	NC_006351.1:230969-231551	100.648	8.31529	-3.5974	-5.19716	0.00035	0.0128852	yes
BPSS0174	-	NC_006351.1:231586-234526	37.7576	1.87794	-4.32955	-4.5343	5.00E-05	0.00324247	yes
BPSS0211	-	NC_006351.1:286129-286321	6443.94	0	-inf	-nan	5.00E-05	0.00324247	yes
BPSS0212	-	NC_006351.1:286413-287052	2485.77	8.30279	-8.22588	-9.92199	0.0001	0.00541923	yes
BPSS0213	-	NC_006351.1:287104-287695	5008.25	12.8831	-8.60268	-10.2979	5.00E-05	0.00324247	yes
BPSS0214	-	NC_006351.1:287773-289249	590.694	13.6792	-5.43236	-5.75792	5.00E-05	0.00324247	yes
BPSS0245	-	NC_006351.1:333658-333886	2270	271.855	-3.06178	-38.7143	0.0005	0.0162463	yes
BPSS0255	-	NC_006351.1:342541-344059	42.4206	6.97219	-2.60508	-3.22817	0.0013	0.0289909	yes
BPSS0256	*rbsC*	NC_006351.1:344067-345045	43.9188	3.24881	-3.75686	-5.04812	0.0011	0.0261486	yes
BPSS0257	-	NC_006351.1:345076-346105	191.942	8.80607	-4.44603	-4.7092	5.00E-05	0.00324247	yes
BPSS0258	-	NC_006351.1:346116-346887	60.3075	3.83501	-3.97503	-5.59195	0.0014	0.0303053	yes
BPSS0259	-	NC_006351.1:346952-348089	103.873	5.85874	-4.14809	-4.85878	5.00E-05	0.00324247	yes
BPSS0327	-	NC_006351.1:456587-457880	447.21	67.3484	-2.73124	-3.24734	0.00255	0.0443077	yes
BPSS0342	-	NC_006351.1:474335-475682	36.4702	6.21684	-2.55247	-3.20169	0.00205	0.0387003	yes
BPSS0369	-	NC_006351.1:508839-509079	4360.76	422.373	-3.36799	-23.3704	0.0012	0.027271	yes
BPSS0409	-	NC_006351.1:549814-566497	220.441	8.91276	-4.62837	-2.61762	0.00235	0.0424506	yes
BPSS0411	-	NC_006351.1:566614-567391	2195.38	21.298	-6.68761	-6.07416	5.00E-05	0.00324247	yes
BPSS0448	-	NC_006351.1:613248-613701	993.947	127.598	-2.96157	-4.65041	0.0008	0.0213045	yes
BPSS0451	-	NC_006351.1:616360-617449	1323.95	161.53	-3.03498	-3.07948	0.0014	0.0303053	yes
BPSS0453	-	NC_006351.1:620792-621200	1808.16	287.004	-2.65538	-5.44525	0.0017	0.0344422	yes
BPSS0493	-	NC_006351.1:670271-671369	258.056	31.166	-3.04964	-3.31293	0.0006	0.0179525	yes
BPSS0546	-	NC_006351.1:746306-747332	411.799	34.7691	-3.56606	-3.87486	0.00025	0.0105579	yes
BPSS0597	-	NC_006351.1:819700-819895	9389.3	1010.45	-3.21603	-176.292	0.00185	0.0363411	yes
BPSS0603	-	NC_006351.1:824838-825141	6413.11	253.762	-4.65948	-13.3074	5.00E-05	0.00324247	yes
BPSS0611	-	NC_006351.1:833781-834690	4877.44	250.249	-4.28469	-4.14477	0.00045	0.0148711	yes
BPSS0612	-	NC_006351.1:834727-836518	2455.33	175.734	-3.80445	-3.40603	0.0012	0.027271	yes
BPSS0614	-	NC_006351.1:837976-840261	508.965	35.3673	-3.84708	-3.8905	0.0004	0.0139317	yes
BPSS0619	*mmsA*	NC_006351.1:844880-846410	255.804	31.2743	-3.03199	-3.24363	0.00105	0.0253488	yes
BPSS0639	-	NC_006351.1:871102-871579	548.647	64.079	-3.09796	-5.2578	5.00E-05	0.00324247	yes

BPSS0666	-	NC_006351.1:902356-904294	129.101	20.9075	-2.6264	-2.47866	0.0011	0.0261486	yes
BPSS0671	-	NC_006351.1:909174-910872	189.719	17.6283	-3.42789	-3.2125	0.00035	0.0128852	yes
BPSS0676	-	NC_006351.1:914573-915398	127.478	21.809	-2.54725	-3.63321	0.00195	0.0376377	yes
BPSS0704	-	NC_006351.1:946181-947270	152.048	3.41999	-5.4744	-5.71236	5.00E-05	0.00324247	yes
BPSS0715	-	NC_006351.1:963138-963672	73.8793	6.89466	-3.42162	-5.1507	0.0015	0.0318473	yes
BPSS0731	-	NC_006351.1:980532-982194	186.813	13.5486	-3.78538	-4.06491	5.00E-05	0.00324247	yes
BPSS0748	-	NC_006351.1:1006474-1006846	1139.45	108.071	-3.39828	-7.29689	0.00045	0.0148711	yes
BPSS0758	-	NC_006351.1:1018535-1019000	1311.88	238.95	-2.45686	-4.39137	0.00255	0.0443077	yes
BPSS0767	-	NC_006351.1:1029558-1029882	2553.37	75.3528	-5.0826	-15.8187	5.00E-05	0.00324247	yes
BPSS0779	-	NC_006351.1:1041218-1042046	374.339	5.34676	-6.12954	-7.34334	5.00E-05	0.00324247	yes
BPSS0781	-	NC_006351.1:1043678-1045535	60.2459	1.42608	-5.40073	-5.55981	0.00045	0.0148711	yes
BPSS0783	-	NC_006351.1:1047506-1048646	55.9001	9.05464	-2.62612	-3.27499	0.0017	0.0344422	yes
BPSS0790	-	NC_006351.1:1055652-1056975	91.0605	2.64222	-5.107	-5.22711	5.00E-05	0.00324247	yes
BPSS0791	-	NC_006351.1:1057246-1058086	13.0587	0	-inf	-nan	5.00E-05	0.00324247	yes
BPSS0813	-	NC_006351.1:1090087-1091503	38.6028	6.5033	-2.56946	-3.01673	0.00125	0.0281595	yes
BPSS0820	-	NC_006351.1:1103627-1107216	468.279	6.35689	-6.2029	-5.43489	5.00E-05	0.00324247	yes
BPSS0826	-	NC_006351.1:1111299-1112457	445.108	63.0064	-2.82059	-3.07661	0.00165	0.0338272	yes
BPSS0849	-	NC_006351.1:1138389-1139310	691.513	45.4134	-3.92857	-4.22959	5.00E-05	0.00324247	yes
BPSS0894	-	NC_006351.1:1186504-1186822	16.9937	0	-inf	-nan	0.00045	0.0148711	yes
BPSS0896	-	NC_006351.1:1187169-1188237	28.7807	4.84515	-2.57049	-2.95704	0.0023	0.04184	yes
BPSS0914	-	NC_006351.1:1208621-1208846	14495.5	598.411	-4.59833	-61.4861	5.00E-05	0.00324247	yes
BPSS0923	-	NC_006351.1:1218569-1219523	5250.36	329.721	-3.9931	-3.92824	0.00085	0.0219936	yes
BPSS0945	-	NC_006351.1:1247072-1248020	202.189	29.9656	-2.75433	-3.71446	0.0006	0.0179525	yes
BPSS0957	-	NC_006351.1:1259880-1261092	121.4	8.17862	-3.89176	-4.92889	5.00E-05	0.00324247	yes
BPSS0958	-	NC_006351.1:1261381-1263367	239.459	26.5787	-3.17143	-2.88883	0.00035	0.0128852	yes
BPSS0959	-	NC_006351.1:1263458-1263899	375.112	49.4168	-2.92425	-5.13134	0.0013	0.0289909	yes
BPSS0960	-	NC_006351.1:1263950-1268576	125.905	13.7513	-3.19469	-2.81732	0.0006	0.0179525	yes
BPSS1045	-	NC_006351.1:1424959-1425448	711.353	82.3696	-3.11038	-5.46533	5.00E-05	0.00324247	yes
BPSS1046	-	NC_006351.1:1425779-1426265	396.316	50.9306	-2.96005	-5.33672	0.00025	0.0105579	yes
BPSS1076	-	NC_006351.1:1447593-1448124	2112.33	44.8508	-5.55756	-6.96391	5.00E-05	0.00324247	yes
BPSS1109	-	NC_006351.1:1487367-1488162	113.825	14.4136	-2.98131	-4.06481	0.00045	0.0148711	yes
BPSS1110	-	NC_006351.1:1488228-1489377	216.885	13.0515	-4.05464	-5.07224	5.00E-05	0.00324247	yes
BPSS1117	-	NC_006351.1:1498962-1499634	308.202	59.054	-2.38377	-3.33514	0.00275	0.0469408	yes
BPSS1156	*narI*	NC_006351.1:1541660-1542344	371.114	51.0054	-2.86314	-3.82033	0.00275	0.0469408	yes

BPSS1199	-	NC_006351.1:1620658-1620922	3833	165.174	-4.53642	-20.2276	5.00E-05	0.00324247	yes
BPSS1226	-	NC_006351.1:1658337-1658616	627.633	83.3995	-2.91181	-12.3983	0.0016	0.0331087	yes
BPSS1229	-	NC_006351.1:1660931-1661714	159.918	6.31071	-4.66338	-6.4987	0.0001	0.00541923	yes
BPSS1230	-	NC_006351.1:1661726-1662884	190.305	10.0208	-4.24724	-5.74231	5.00E-05	0.00324247	yes
BPSS1231	-	NC_006351.1:1663299-1663770	377.767	69.5498	-2.44138	-4.60672	0.0024	0.0426089	yes
BPSS1282	-	NC_006351.1:1751485-1752982	83.0746	12.4918	-2.73343	-3.29492	0.00055	0.0171174	yes
BPSS1286	-	NC_006351.1:1757582-1757801	6283.86	1141.68	-2.46049	-43.685	0.00205	0.0387003	yes
BPSS1312	-	NC_006351.1:1797051-1798512	199.272	26.7323	-2.89809	-3.04112	0.0002	0.00888195	yes
BPSS1360	-	NC_006351.1:1860466-1862680	258.802	22.6764	-3.51258	-3.50507	0.0001	0.00541923	yes
BPSS1453	-	NC_006351.1:1980961-1985591	339.264	18.8859	-4.16703	-3.19245	0.00045	0.0148711	yes
BPSS1455	-	NC_006351.1:1985592-1986384	506.54	23.0138	-4.46011	-5.465	5.00E-05	0.00324247	yes
BPSS1555	-	NC_006351.1:2109378-2111076	1464.89	86.2226	-4.08658	-3.25451	0.00045	0.0148711	yes
BPSS1647	-	NC_006351.1:2262605-2264946	82.4235	4.64252	-4.15007	-2.93659	0.0014	0.0303053	yes
BPSS1652	-	NC_006351.1:2270440-2271439	47.2362	6.43526	-2.87582	-3.57121	0.001	0.0247193	yes
BPSS1658	-	NC_006351.1:2280112-2280697	2183.83	14.8031	-7.20482	-9.33165	5.00E-05	0.00324247	yes
BPSS1731	-	NC_006351.1:2377102-2378716	151.575	4.54916	-5.05829	-4.35084	5.00E-05	0.00324247	yes
BPSS1734	-	NC_006351.1:2381112-2382861	1836.75	11.7759	-7.28518	-5.96218	5.00E-05	0.00324247	yes
BPSS1735	-	NC_006351.1:2382955-2383795	204.544	22.4751	-3.18601	-4.35621	0.00025	0.0105579	yes
BPSS1740	*lipB*	NC_006351.1:2389151-2390186	360.486	45.884	-2.97388	-4.13816	0.00035	0.0128852	yes
BPSS1741	*lipA1*	NC_006351.1:2390189-2391284	1611.06	47.0198	-5.09859	-4.76068	5.00E-05	0.00324247	yes
BPSS1753	-	NC_006351.1:2403908-2404769	326.827	27.9558	-3.54731	-3.19132	0.0001	0.00541923	yes
BPSS1797	-	NC_006351.1:2456108-2457752	545.377	53.8188	-3.34107	-3.76513	0.00055	0.0171174	yes
BPSS1801	-	NC_006351.1:2461062-2461500	150.27	18.9244	-2.98924	-5.24415	0.00165	0.0338272	yes
BPSS1802	-	NC_006351.1:2461741-2462368	475.462	56.7342	-3.06704	-4.0725	0.0004	0.0139317	yes
BPSS1805	-	NC_006351.1:2465124-2465709	138.357	11.287	-3.61566	-5.45422	5.00E-05	0.00324247	yes
BPSS1812	-	NC_006351.1:2470643-2471441	100.803	12.1622	-3.05107	-4.19493	0.00015	0.00726516	yes
BPSS1813	-	NC_006351.1:2471444-2472350	636.953	24.0124	-4.72934	-4.96523	5.00E-05	0.00324247	yes
BPSS1815	-	NC_006351.1:2472446-2474293	61.6488	7.02144	-3.13423	-2.69655	0.0003	0.0117121	yes
BPSS1837	-	NC_006351.1:2499155-2499737	484.765	20.8461	-4.53944	-6.14439	5.00E-05	0.00324247	yes
BPSS1838	-	NC_006351.1:2499762-2501100	378.404	8.26673	-5.51647	-5.98897	5.00E-05	0.00324247	yes
BPSS1839	-	NC_006351.1:2501207-2501630	3349.44	41.3946	-6.33833	-10.9285	5.00E-05	0.00324247	yes
BPSS1844	-	NC_006351.1:2505911-2506265	1995.39	296.332	-2.75138	-6.88401	0.0008	0.0213045	yes
BPSS1891	*catB*	NC_006351.1:2566793-2567927	646.611	25.6736	-4.65454	-4.78509	5.00E-05	0.00324247	yes
BPSS1892	*catA*	NC_006351.1:2567961-2568864	532.173	12.016	-5.46887	-6.49552	5.00E-05	0.00324247	yes

BPSS1893	*catC*	NC_006351.1:2568901-2569192	854.248	47.5476	-4.16721	-14.3973	0.0001	0.00541923	yes
BPSS1894	*cyoD*	NC_006351.1:2569311-2569644	431.235	62.0459	-2.79706	-8.1012	0.00085	0.0219936	yes
BPSS1911	-	NC_006351.1:2588239-2589724	564.151	31.5355	-4.16103	-4.01239	5.00E-05	0.00324247	yes
BPSS1979	-	NC_006351.1:2677446-2678637	57.5818	5.03057	-3.51682	-3.78364	5.00E-05	0.00324247	yes
BPSS1983	-	NC_006351.1:2681881-2682934	454.151	8.32938	-5.76882	-5.72899	5.00E-05	0.00324247	yes
BPSS1984	-	NC_006351.1:2683005-2686691	233.212	4.81281	-5.59862	-4.72664	5.00E-05	0.00324247	yes
BPSS1986	-	NC_006351.1:2686734-2687490	376.542	7.79561	-5.59401	-7.47192	5.00E-05	0.00324247	yes
BPSS1987	-	NC_006351.1:2687490-2690126	447.617	17.5076	-4.67621	-3.49863	0.0007	0.019907	yes
BPSS1989	-	NC_006351.1:2690148-2691303	611.295	10.1513	-5.91213	-6.47948	5.00E-05	0.00324247	yes
BPSS1999	-	NC_006351.1:2706194-2707031	280.434	3.77542	-6.21488	-7.28891	0.0006	0.0179525	yes
BPSS2001	-	NC_006351.1:2708346-2708595	9993.15	141.337	-6.14373	-36.394	5.00E-05	0.00324247	yes
BPSS2021	-	NC_006351.1:2731209-2732691	121.867	21.849	-2.47966	-2.91476	0.00135	0.0298483	yes
BPSS2022	-	NC_006351.1:2732830-2733736	1386.45	70.679	-4.29397	-4.02273	5.00E-05	0.00324247	yes
BPSS2024	-	NC_006351.1:2734374-2735583	70.0271	7.39564	-3.24317	-3.85907	0.00015	0.00726516	yes
BPSS2032	-	NC_006351.1:2745510-2746662	50.2698	4.3063	-3.54517	-3.82112	0.00015	0.00726516	yes
BPSS2034	-	NC_006351.1:2746668-2749520	13.6823	1.81246	-2.91629	-2.58568	0.0021	0.0393566	yes
BPSS2035	-	NC_006351.1:2749554-2750439	27.3565	3.01428	-3.182	-3.60851	0.0024	0.0426089	yes
BPSS2036	-	NC_006351.1:2750801-2752463	30.2459	4.09508	-2.88477	-3.1868	0.00055	0.0171174	yes
BPSS2037	-	NC_006351.1:2753571-2754483	364.502	3.64744	-6.6429	-7.58432	5.00E-05	0.00324247	yes
BPSS2039	-	NC_006351.1:2754805-2756778	1080.27	25.6418	-5.39675	-4.93701	5.00E-05	0.00324247	yes
BPSS2043	-	NC_006351.1:2759707-2760127	2310.12	250.765	-3.20356	-5.77096	0.0004	0.0139317	yes
BPSS2044	-	NC_006351.1:2760199-2761105	576.406	54.3047	-3.40794	-4.14984	0.00035	0.0128852	yes
BPSS2084	-	NC_006351.1:2820341-2821583	83.6249	8.35699	-3.32288	-3.24525	0.0001	0.00541923	yes
BPSS2086	-	NC_006351.1:2822905-2824966	93.3537	14.2874	-2.70797	-2.48636	0.0011	0.0261486	yes
BPSS2098	-	NC_006351.1:2839990-2840482	80.3592	5.75786	-3.80286	-5.41793	0.002	0.0381749	yes
BPSS2104	-	NC_006351.1:2847579-2851209	34.8483	3.42388	-3.34738	-3.32548	0.0004	0.0139317	yes
BPSS2106	-	NC_006351.1:2852543-2853932	47.0721	3.49866	-3.75	-4.03868	0.00015	0.00726516	yes
BPSS2214	*katE*	NC_006351.1:2988111-2990238	31.5662	4.36396	-2.85467	-2.93791	0.0008	0.0213045	yes
BPSS2219	-	NC_006351.1:2993472-2994231	53.8475	8.01022	-2.74897	-3.55574	0.0012	0.027271	yes
BPSS2265	-	NC_006351.1:3042680-3044030	434.077	18.9925	-4.51445	-4.69031	5.00E-05	0.00324247	yes
BPSS2266	-	NC_006351.1:3044310-3045126	414.325	32.3153	-3.68047	-5.19437	5.00E-05	0.00324247	yes
BPSS2267	-	NC_006351.1:3045343-3046597	1047.66	39.8387	-4.71685	-5.73406	5.00E-05	0.00324247	yes
BPSS2268	-	NC_006351.1:3046959-3047433	211.226	28.0268	-2.91391	-5.41936	0.00085	0.0219936	yes
BPSS2270	*lpdV*	NC_006351.1:3050149-3051550	758.296	11.3365	-6.06371	-7.19354	5.00E-05	0.00324247	yes

BPSS2271	*bkdB*	NC_006351.1:3051554-3053006	512.28	13.3919	-5.2575	-5.81762	5.00E-05	0.00324247	yes
BPSS2272	*bkdA2*	NC_006351.1:3053007-3054051	1551.93	25.7038	-5.91594	-6.11401	5.00E-05	0.00324247	yes
BPSS2273	*bkdA1*	NC_006351.1:3054056-3055289	1090.24	25.4219	-5.42243	-5.26559	5.00E-05	0.00324247	yes
BPSS2274	-	NC_006351.1:3056540-3056981	3565.82	97.3119	-5.19547	-8.26469	5.00E-05	0.00324247	yes
BPSS2275	-	NC_006351.1:3057305-3058421	991.065	52.511	-4.23829	-5.01443	5.00E-05	0.00324247	yes
BPSS2276	-	NC_006351.1:3058718-3059654	538.616	70.293	-2.9378	-3.22291	0.0022	0.0405926	yes
BPSS2277	-	NC_006351.1:3059828-3060260	772.308	95.9849	-3.0083	-5.52074	0.00045	0.0148711	yes

Functional categories of genes overexpressed in bacteria grown in plasma: energy metabolism (31 genes)

Gene	Common Gene Name	Description	KEGG Pathway	FPKM value Plasma	Soil	Log_2 Fold Change
**Carbohydrate and lipid metabolism**						
BPSL0687	*glpK*	glycerol kinase	Glycerolipid meta	130.695	21.94	2.57457
BPSL0688	*glpD*	glycerol-3-phosphate dehydrogenase	Glycerophospholi	253.162	20.2263	3.64576
BPSL2299	*BPSL2299*	putative dihydrolipoamide dehydroger	Glycolysis / Gluco	576.842	53.3781	3.43386
BPSL2300	*pdhB*	dihydrolipoamide acetyltransferase	Glycolysis / Gluco	496.155	35.8795	3.78956
BPSL2932	*edd*	phosphogluconate dehydratase	Pentose phospha	283.608	25.3862	3.48178
BPSS0144	*BPSS0144*	amylase	Starch and sucros	214.172	17.4027	3.62138
BPSS0840	*BPSS0840*	zinc-binding dehydrogenase	Glycolysis / Gluco	193.254	6.57764	4.87679
BPSS1582	*bcsB*	cellulose synthase regulator protein		17.6274	0.763976	4.52815
BPSS1918	*BPSS1918*	alcohol dehydrogenase	Glycolysis / Gluco	121.146	11.8561	3.35304
**Oxidative phosphorylation**						
BPSL0502	*cydA*	cytochrome d ubiquinol oxidase subun	Oxidative phosph	1022.78	65.8252	3.95771
BPSS0234	*BPSS0234*	cytochrome oxidase subunit II	Oxidative phosph	406.918	3.18387	6.99781
BPSS0235	*BPSS0235*	cytochrome oxidase subunit I	Oxidative phosph	535.001	8.39457	5.99394
BPSS1949	*BPSS1949*	ATP synthase A chain	Oxidative phosph	121.545	6.54352	4.21528
BPSS1953	*atpD*	F0F1 ATP synthase subunit beta	Oxidative phosph	339.963	2.25939	7.2333
**Nitrogen metabolism**						
BPSL2309	*narG*	respiratory nitrate reductase alpha cha	Nitrogen metabo	484.346	6.3671	6.24926
BPSL2312	*BPSL2312*	putative respiratory nitrate reductase g	Nitrogen metabo	352.479	8.23467	5.41968
BPSS1241	*BPSS1241*	bifunctional reductase	Nitrogen metabo	202.428	5.82673	5.11858
BPSS1243	*nirB*	nitrite reductase (NAD(P)H)	Nitrogen metabo	675.161	16.0078	5.39838
**Arginine deiminase system**						
BPSL1742	*arcD*	arginine/ornithine antiporter		726.922	4.97384	7.19129
BPSL1743	*arcA*	arginine deiminase	Arginine and prol	1125.59	3.08151	8.51283
BPSL1744	*arcB*	ornithine carbamoyltransferase	Urea cycle and m	1604.21	5.23491	8.25948

BPSL1745	*arcC*	carbamate kinase	Purine metabolisı	1472.96	10.0371	7.19723
**Phenylacetic acid catabolism pathway**						
BPSL3232	*paaC*	phenylacetic acid degradation protein PaaC		285.419	17.8857	3.9962
BPSL3233	*paaD*	phenylacetic acid degradation protein PaaD		90.6275	4.23556	4.41932
BPSL3234	*paaE*	probable phenylacetic acid degradation NADH oxidoredu		205.842	13.5574	3.92439
**Other**						
BPSS0103	*BPSS0103*	hypothetical protein		39.7657	3.80037	3.38731
BPSS0481	*BPSS0481*	CoA ligase	Carbazole degrad	38.4409	6.03658	2.67084
BPSS0551	*BPSS0551*	hypothetical protein		124.333	8.4886	3.87254
BPSS0552	*BPSS0552*	electron transfer flavoprotein alpha-subunit		103.633	4.59282	4.49596
BPSS0554	*BPSS0554*	iron-sulphur Rieske protein		185.843	8.84655	4.39282
BPSS0721	*BPSS0721*	enoyl-(acyl carrier protein) reductase	Fatty acid biosynt	82.4135	5.69475	3.85518

Functional categories of genes overexpressed in bacteria grown in plasma: cullular metabolism (30 genes)

Gene	Common Gene Name	Description	KEGG Pathway	FPKM value Plasma	FPKM value Soil	Log$_2$ Fold Change
**Flagellar assembly**						
BPSL0030	*fliP*	flagellar biosynthesis protein FliP	Flagellar assembly; Type III secretion system	181.733	18.9217	3.2637
BPSL0269	*flgA*	flagellar basal body P-ring biosynthesis protein FlgA	Flagellar assembly	97.1323	11.6859	3.05519
BPSL0274	*flgF*	flagellar basal body rod protein FlgF	Flagellar assembly	382.101	44.012	3.11798
BPSL0275	*flgG*	flagellar basal body rod protein FlgG	Flagellar assembly	571.197	47.8823	3.57643
BPSL0276	*flgH*	flagellar basal body L-ring protein	Flagellar assembly	272.268	21.4037	3.66909
BPSL0277	*flgI*	flagellar basal body P-ring protein	Flagellar assembly	265.561	30.361	3.12875
BPSL0280	*flgK*	flagellar hook-associated protein FlgK	Flagellar assembly	960.499	84.6923	3.50348
BPSL0281	*flgL*	flagellar hook-associated protein FlgL	Flagellar assembly	1625.36	134.294	3.5973
BPSL3320	*fliD*	flagellar hook-associated protein	Flagellar assembly	953.486	92.44	3.36662
**Type III secretion system**						
BPSS1534	*bsaZ*	surface presentation of antigens protein SpaS	Type III secretion system	83.1711	5.66845	3.87506
BPSS1535	*bsaY*	surface presentation of antigens protein	Type III secretion system	73.853	5.03283	3.87521
BPSS1543	*bsaQ*	Type III secretion system protein	Type III secretion system	116.264	4.08162	4.83212
BPSS1545	*bsaO*	Type III secretion system protein	Type III secretion system	290.707	5.38728	5.75386
BPSS1546	*bsaN*	AraC-family regulator of type III secretion system		453.924	6.62593	6.09818
BPSS1550	*BPSS1550*	Type III secretion system protein		254.244	5.59784	5.5052
**Two-component system and bacterial chemotaxis**						

BPSL2367	*BPSL2367*	putative methyl-accepting chemotaxis protein	Two-component system; Bacterial chemotaxis	257.097	13.8129	4.21822
BPSL3300	*cheY*	chemotaxis protein CheY	Two-component system; Bacterial chemotaxis	919.899	150.423	2.61245
BPSS0276	*aer*	aerotaxis receptor	Two-component system; Bacterial chemotaxis	269.315	6.328	5.4114
BPSL0465	*BPSL0465*	putative methyl-accepting chemotaxis protein	Bacterial chemotaxis	154.678	21.7065	2.83307
BPSS1997	*oxa*	beta-lactamase precursor	Penicillin and cephalosporin biosynthesis; Two-component system	1052.81	33.6993	4.96538
**Universal stress family protein**						
BPSS1140	*BPSS1140*	universal stress family protein		1278.22	107.893	3.56646
BPSS1934	*BPSS1934*	universal stress protein		1675.37	21.5142	6.28304
**Other**						
BPSL0328	*BPSL0328*	putative 2-nitropropane dioxygenase	Nitrogen metabolism	472.894	83.5509	2.50079
BPSL1804	*amrA*	periplasmic multidrug efflux lipoprotein precursor		115.099	12.1568	3.24304
BPSL2778	*BPSL2778*	putative tyrosine-protein kinase involved in EPS biosynthesis		12.4089	1.90446	2.70393
BPSS0839	*BPSS0839*	hypothetical protein		509.514	16.5141	4.94735
BPSS1043	*czcC*	cobalt-zinc-cadmium resistance protein		28.2158	2.76101	3.35324
BPSS1354	*betB*	betaine aldehyde dehydrogenase	Glycine, serine and threonine metabolism	623.178	18.5376	5.07112
BPSS1355	*betA*	choline dehydrogenase	Glycine, serine and threonine metabolism	381.424	26.514	3.84657
BPSS2288	*BPSS2288*	HSP20/alpha crystallin family protein		2057.76	63.0735	5.0279
